# Urban pollution in the Danube and Western Balkans regions: The impact of major PM_2.5_ sources

**DOI:** 10.1016/j.envint.2019.105158

**Published:** 2019-12

**Authors:** Claudio A. Belis, Enrico Pisoni, Bart Degraeuwe, Emanuela Peduzzi, Philippe Thunis, Fabio Monforti-Ferrario, Diego Guizzardi

**Affiliations:** a European Commission, Joint Research Centre, via Fermi, 2749, 21027 Ispra, Italy; b Didesk Informatica, Verbania, Italy

## Abstract

The SHERPA tool was used to assess the major pollution sources and the geographical areas impacting on the PM_2.5_ of the main cities in the Danube and Western Balkans regions. The activity sectors influencing most the PM_2.5_ levels in the study area are energy production (22%), agriculture (19%), residential combustion (16%) and road transport (7%). The energy production in inefficient coal-fuelled power plants was identified as one of main source of PM_2.5_ in the Western Balkans. As for the geographical origin of PM_2.5_, the transboundary pollution is confirmed as the main origin of PM_2.5_ (44%) in the investigated cities, while the city own emissions and the national sources outside the concerned city impact on average 22% and 15%, respectively. An association was observed between the long-range transport and the impact of agriculture and energy production, while both local urban emissions and long-range transport were associated with the residential sector. A special attention is given in this study to biomass, a renewable source, which use is often promoted in the frame of climate and energy policies. Nevertheless, the combustion of biomass in inefficient small appliances has considerable particulate matter emissions and therefore this type of practice impacts negatively on air quality. Considering that biomass is traditionally used in South-East Europe as fuel for residential heating, the interpretation of the model results was supported with the estimation of biomass burning contributions to PM_2.5_ obtained with receptor models and data on biomass fuel consumption from the literature. The analysis of the contributions from biomass burning derived from receptor models suggests that biomass burning is the dominant source within the residential heating sector in the studied area and that the emissions from this source are likely underestimated. This study concludes that more effort is needed to improve the estimations of biomass burning emissions and that policies to improve air quality in the cities should involve a geographic context wider than the city level.

## Highlights

• The SHERPA tool based on EMEP model and emissions was used for source quantification
• The residential sector impact was compared with biomass burning receptor model data
• Coal-fuelled power plants are among the main sources of PM_2.5_ in the Western Balkans
• Biomass burning is the dominant source within the residential heating sector
• Transboundary pollution is associated with agriculture and energy sectors

## 1 Introduction

Air pollution is the main environmental cause of premature death. Ambient air pollution in cities and rural areas caused 4.2 million deaths worldwide in 2016 contributing together with indoor pollution to 7.6% of all deaths (WHO, 2018). In the cited report, most countries from the Danube and Western Balkans regions, i.e. Bosnia and Herzegovina, Bulgaria, Montenegro, Serbia, Romania and North Macedonia are listed among those with the highest mortality attributed to household and ambient air pollution in Europe. Overall, premature deaths and morbidity caused by air pollution in Europe (UNECE region) have an estimated burden for the community worth EUR 1000 billion and EUR 100 billion, respectively (WHO Regional Office for Europe and OECD, 2015).

Particulate matter (PM) is one of the most common and harmful atmospheric pollutants. PM_10_ (PM with an aerodynamic diameter ≤ 10 μm) is responsible for respiratory effects due to short-term exposure while PM_2.5_, the finest fraction (PM ≤ 2.5 μm), is associated with mortality due to long-term exposure (WHO Regional Office for Europe, 2013). Different cohort studies in Europe and North America concluded there is an increased risk of cardiopulmonary mortality of 6–13% per 10 μg/m^3^ of PM_2.5_ (Beelen et al., 2008; Krewski et al., 2009; Pope III and Dockery, 2006). More recently, the HRAPIE (Health Risks of air pollution in Europe) project suggested a Relative Risk of 1.062 with respect to all-cause (natural) mortality in adult populations per 10 μg/m^3^ of long-term exposure to PM_2.5_ (Health risks of air pollution in Europe - HRAPIE project, 2013), based on the meta-analysis by Hoek et al. (2013).

Levels of air pollutants often exceed the limit values of PM_10_ in many zones of South-East Europe leading to infringement procedures to the EU member states of this area. In non-EU member states the information about air pollution levels is improving thanks to the development of the monitoring networks (EEA, 2018). Nevertheless, there are still gaps in the time and spatial coverage for PM_10_ and even more for PM_2.5_, as the monitoring of the latter started more recently than the former and the number of monitoring sites is lower.

A simulation of the atmospheric pollution in the entire Balkan region using the US-EPA Model-3 system (MM5, CMAQ and SMOKE) based on a TNO emission inventory for the reference year 2003 has associated the levels of NO_2_, PM_2.5_ and coarse PM with the energy production and road transport (Gadzhev et al., 2011). According to EMEP emission estimations for 2014 elaborated by the JRC (Belis et al., 2017), combustion in the domestic sector, industry and public power plants are to varying extents the main emission sources of PM_10_ and PM_2.5_ in countries of the Eastern Balkan Peninsula (Croatia, Serbia, Slovenia, Albania and Montenegro). Studies with receptor models showed that primary emissions from traffic, biomass burning together with secondary processes were those contributing more to the PM_10_ and PM_2.5_ in a number of urban areas of the Danube region (Zagreb, Sofia, Budapest, Belgrade, Banja Luka, Chrisinau, Nikišić) (Perrone et al., 2018; Almeida et al., 2019). In addition, the influence of other sources, such as industrial emissions and those deriving from the combustion of fuel oil, varied considerably from site to site. The first of these studies also concluded that most of the secondary aerosol and soil resuspension (relevant for PM_10_) in the studied cities are subject to long-range transport from areas outside the EU, and therefore not able to be abated with the EU policies alone (Perrone et al., 2018).

Central and Eastern Europe are the European regions with the highest proportion of PM_2.5_ emissions deriving from residential heating with solid fuels (21% and 13%, respectively in 2010; WHO Regional Office for Europe, 2015). In South-East Europe, biomass is traditionally used as fuel for residential heating because of its accessibility and low costs, especially in rural areas. Since the combustion of biomass in outdated small appliances has considerable emissions of particulate matter, black carbon and PAHs, this type of source impacts negatively on air quality and, consequently on human health (Li et al., 2017). Furthermore, some studies have hypothesised that the toxicity of particles may vary depending on their composition and sources (Tuomisto et al., 2008; Lelieveld et al., 2015). Nevertheless, considering that biomass is an important source of renewable carbon-neutral energy source (although not always fully carbon-neutral), its use is generally promoted by the climate and energy policies. Therefore, the biomass use for energy and heat generation, and consequently the consumption of this fuel in the Danube region, is expected to increase by 38% in 2020 with respect to 2013 (Banja et al., 2017; Monforti-Ferrario and Belis, 2018).

The objectives of this work is to identify the activity sources and the geographical areas (spatial sources) contributing to PM_2.5_ concentrations in the main cities of the Danube and Western Balkans (WB) regions with particular reference to the impact of biomass burning in the residential sector. To that end, the SHERPA screening tool based on the EMEP model was run for the base year 2014 and the results integrated with estimations obtained with receptor models and statistical information about the use of fuels from the literature. The used tool, the underlying model and the study area are illustrated in chapter 2, the PM levels are described in chapter 3.1, the sources' impacts on PM_2.5_ in the study area are presented in chapter 3.2, the role of biomass burning in the residential sector is analysed in chapter 3.3 while discussion and conclusions of the study are reported in chapters 4 and 5, respectively.

## 2 Methodology

### 2.1 Sherpa tool based on EMEP model

Chemistry transport models (CTM) simulate the dispersion processes and chemical reactions to calculate the concentration of pollutants using as input the emission inventories, meteorological fields and boundary conditions. These models are complex and require considerable computational resources. The SHERPA (Screening for High Emission Reduction Potential on Air) tool was developed by the JRC to compute and explore the levels of pollutants in a short time using limited computational resources (Clappier et al., 2015; Thunis et al., 2016; Pisoni et al., 2017).

The SHERPA methodology is based upon the concept of “Geographically Weighted Regression “(GWR, Fotheringham et al., 2002; Oshan and Fotheringham, 2017; Wolf et al., 2018) or “local modelling approaches” (Lloyd, 2010), a family of techniques that use “bell-shaped” kernel functions to establish weighted, local regressions between input and output variables. In practice, SHERPA simulates the link between input (“change in emissions in comparison to the base case”) and output (“change in concentrations in comparison to the base case”), using data from more complex/deterministic models. More specifically, the SHERPA tool is defined through statistical relations: a set of cell-dependent coefficients known as source receptor relationships (SRR), trained and validated using data coming from a CTM run for a specific set of simulations. SHERPA implements a source apportionment approach known as brute force or emission reduction impact (Thunis et al., 2019; Belis et al., 2019). A more detailed description of the SHERPA methodology can be found in Pisoni et al. (2017).

The SHERPA methodological approach adopted in this study is identical to the one described in Thunis et al. (2018) with the only difference that the SRRs were computed based on the EMEP MSC-W chemical transport air quality model (hereon EMEP model; Simpson et al., 2012). This model was selected because is used by the EMEP modelling centre on photo-oxidants and aerosol to support the Convention on Long Range Transboundary Air Pollution (CLRTAP) and is the basis for the air quality estimations of the Greenhouse gas - Air pollution Interactions and Synergies model (GAINS) scenario analysis. The EMEP model domain encompasses the entire continental Europe until 37° E, the British Isles and the Mediterranean area until 32° N. Such a large extension including parts of Eastern Europe and Northern Africa is particularly suitable to account for the transboundary pollution originated either outside the study area and beyond the EU borders.

The EMEP model vertical structure is made of 20 layers the lowermost of which is approx. 45 m height above ground level. The convective mass flux scheme was based on Tiedtke (1989). The meteorological fields were derived from PARLAM-PS, HIRLAM and ECMWF-IFS numerical weather prediction models. The emission input was provided by the EMEP land-based gridded emissions database for 2014 structured according to the Selected Nomenclature for sources of Air Pollution (SNAP) codes. The EMEP model used a lumped molecule approach for VOC split in 14 species (Simpson et al., 2012; Table S5). Modelled sulphate was obtained by oxidation of SO_2_ both in the gas and the aqueous phases assuming equilibrium between them. For the aqueous phase reactions it was assumed that nitrate, sulphate and HNO_3_ were completely dissolved and that the Henry's law was fulfilled. The only considered oxidants were O_3_, H_2_O_2_ and O_2_. The pH in cloud water was estimated from the acid-base balance. In the gas phase, SO_2_ was oxidised both in the cloud free parts of the grid box and in the interstitial cloud air by a chain of reactions initiated by the OH radical. The HNO_3_ formed by the reaction of N_2_O_5_ with water was assumed to evaporate and take part in the formation of ammonium nitrate. The N_2_O_5_ reaction rate was computed as a function of aerosol surface area and composition. The effect of relative humidity (> 40%) on the particles surface and density, and consequently on the rate of heterogeneous reactions, was accounted for.

For the secondary organic aerosol (SOA) a simplified version of the VBS approach (Bergström et al., 2012) was used (NPAS scheme). In such scheme all the primary organic aerosol (POA) emissions are assumed to be non-volatile while the semi-volatile SOA are assumed to be oxidised by OH-reactions leading to increased partitioning to the solid phase. The emissions from international shipping accounted for reduced sulphur emissions in the years preceding 2014 while emissions from national shipping were not included. Biogenic emissions of isoprene and monoterpenes were calculated using near-surface air temperature and photosynthetically active radiation. The forest species were parameterised on the basis of aggregated land-cover types' maps. The model accounted for contributions from natural mineral dust resulting from resuspension of dust from deserts, semi-arid areas, agricultural and bare lands within the model domain, as well as dust produced beyond the model grid and transported to the calculation domain. A simplified road-dust module has also been implemented (Simpson et al., 2012).

In the SHERPA tool, the EMEP total emissions per sector, country and pollutants for 2014 were gridded using the JRC derived proxies, for the EU28 domain, (Trombetti et al., 2017) and applying EMEP own gridding methodology for the countries outside the EU 28 domain (EMEP/CEIP, 2014). A study on the comparability of different emission inventories (Trombetti et al., 2018) has shown that the two gridding methodologies used in the present study are coherent and, therefore, the comparison among cities is not hindered.

Because of its simplifying assumptions and the relatively coarse spatial resolution of the EMEP model (0.1 by 0.1° which corresponds to approximately 8 km longitude by 11 km latitude in the Western Balkans), SHERPA is best indicated to compute yearly average concentration levels of background PM_2.5_ for relatively large cities. For that reason, in this study only cities of the Danube and WB regions with >100,000 inhabitants have been investigated.

The delimitation of the urban areas studied in this work is based on the Functional Urban Areas (FUA) concept which includes the core city plus the wider commuting zone defined as the surrounding travel-to-work areas where at least 15% of the employed residents work in the city (OECD, 2012). In order to include the cities of the Western Balkans that are not part of the EU28, and consequently not present in the OECD urban datasets, it was necessary to define the shape of the FUA for 13 cities in Albania, Bosnia and Herzegovina, Kosovo, Montenegro, Serbia and North Macedonia. This was carried out using the remote sensing data and census data processed by the Global Human Settlement Layer (GHSL) project of the European Commission, Joint Research Centre (Corbane et al., 2017; https://ghsl.jrc.ec.europa.eu/). The areas of the Western Balkans' FUAs are shown in Fig. S1 of the Supplementary material.

In this study, the SHERPA tool is used to assess both the impact of the main activity sources on PM_2.5_ and their geographical origin. The PM_2.5_ emitting sources are classified according to the SNAP macro-sectors (EEA, 1996). The categories defined to describe the geographical origin of pollution are: a) FUA (see explanation above); b) national, including the rest of the emissions in the country, and c) international, representing the emission from the entire model domain, with the exception of the country itself. A fourth category, external, includes the emissions outside the domain and those not allocated to a specific source (e.g. international maritime traffic).

### 2.2 Model validation and limitations of the used approach

#### 2.2.1 Validation of the EMEP model

**Fig. 1:**
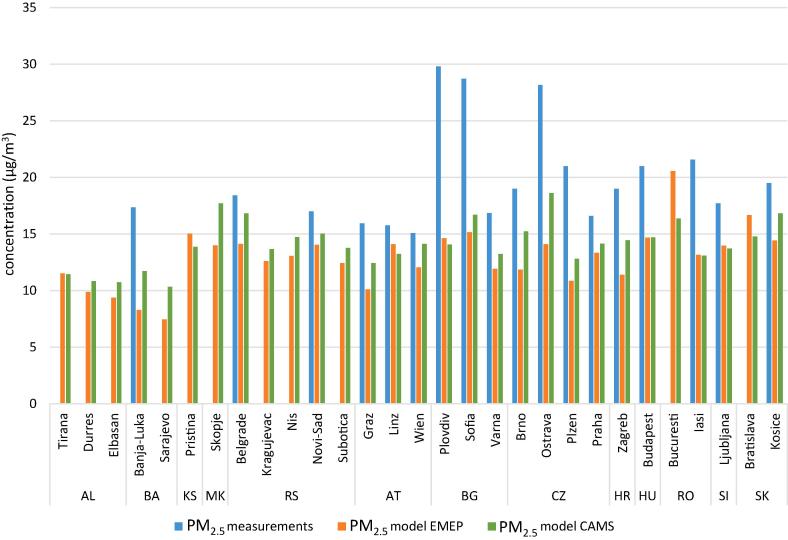
Modelled PM_2.5_ concentrations for some Danube and Western Balkans cities using the EMEP model (this study) and the CAMS ensemble model compared with the measured concentrations (source: European Environment Agency, Serbian Environment Protection Agency and Hydrometeorological Service of Republic of Srpska, Bosnia and Herzegovina).

The agreement between model results and observations depends on several factors. The accuracy of the measurements and the representativeness of the measurement sites, on one hand and the reliability of the input data (emissions, boundary conditions and meteorological fields) and the model performance on the other hand. Therefore, lack of correlation between the two set of data is not necessarily an indication of poor model performance. The validation of the EMEP model was carried out using 41 and 31 (for PM_10_ and PM_2.5_, respectively) EMEP monitoring station distributed across Europe and the results are summarised in Table S1 of the Supplementary material (EMEP, 2016). The overall PM_2.5_ annual mean underestimation for 2014 was 13%. The only chemical components tested for PM_2.5_ were sulphate (−24%), EC (−22%) and OC (−55%). Four of the monitoring stations used for the validation are located in the domain of this study (Illmitz, AT; Kosetice, CZ; K-puszta, HU and Iskrba, SI). The PM_2.5_ bias for these stations ranged between −18% and − 35% (EMEP, 2016). Unfortunately there is very limited information on PM_2.5_ for 2014 in the Western Balkans. In Fig. 1 are compared the measured PM_2.5_ in urban background sites (reported in AIRBASE/EEA AQ database complemented with data from some monitoring networks) with the results of the EMEP model used for this study. The EMEP model negative bias in this study area (−33%) is considerably higher than the one observed for the entire Europe (−13%). The CAMS ensemble model (https://atmosphere.copernicus.eu/) is based on the median values of seven state-of-the-art numerical air quality models (CHIMERE, EMEP, EURAD-IM, LOTOS-EUROS, MATCH, MOCAGE and SILAM). The difference between measurements and CAMS ensemble model PM_2.5_ estimations for 2014 is comparable, in terms of bias (−25%) and geographical pattern, to the one observed for the EMEP model. The similarity between EMEP and CAMS ensemble (only 8% difference) suggests that the strong underestimations of PM_2.5_ in this area are not to be attributed to the air quality model only and are likely also due to other factors such as the emission input data.

#### 2.2.2 Validation of SHERPA

SHERPA links the change in emissions (in comparison to the base case) to its correspondent change in concentrations of the target pollutant (PM_2.5_ in this case) based on the ‘signal’ resulting from the Chemical Transport Model simulation (EMEP in this case). Since SHERPA does not reproduce the base case simulation its validation is made in two steps:

- Test base case simulation by comparing the CTM simulation with the available observations (see previous paragraph).
- Test the emission reduction scenario simulations by comparing the CTM emission reduction scenario results (i.e. reducing European or regional emissions, or reducing emissions per precursor or sector) and the correspondent SHERPA signal.

The results of the second step validation are shown in Fig. S2 of the Supplementary material for 13 cities of the European domain. In the city of Sofia, that has been used as representative for SE Europe, the median percentage bias for all the tested pollutant precursors are close to zero and their distribution fall, with few exceptions within the interval ± 5%. The results of the test for the city of Sofia are comparable to those of the other tested cities.

#### 2.2.3 Limitations of the EMEP model

The height of the lowermost layer may impair the ability of the model to estimate vertical dispersion under strong winter thermal inversion conditions (this is relevant for biomass burning emissions). However, modelling stable wintertime surface layer is problematic for all the models at the synoptic scale. Uncertainties are also associated with the estimation of the particulate nitrate (Simpson et al., 2012). The parameterisation for the uptake rate of N_2_O_5_ may vary considerably due to influence of a number of factors, such as the concentration of organic matter (OM). In addition, the enhancing effect of OM and other fine PM on aerosol surface and the inhibiting effect of OM on the particle sticking coefficient were not taken into account in this run. One of the main limitations of the used VOC approach is that the distribution of POA emissions into semi- (SVOC) and intermediate- (IVOC) volatile organic compounds is poorly known as there is limited detail in the emission inventories about these fractions. Sensitivity analysis showed that the application of the NPAS scheme led to PM_2.5_ total year average OA concentrations 10–20% lower than a scheme where SVOC and IVOC are accounted for (Simpson et al., 2012). Finally, it should be considered that the spatial resolution of the model (0.1 × 0.1°) is not suitable to detect urban hot spots due to local sources such as traffic.

#### 2.2.4 Limitations associated to the SHERPA tool

The uncertainty of the SHERPA output depends on limitations associated with the underlying air quality model, the input data and the SHERPA tool assumptions. The emission inventory strongly influences the EMEP model and consequently the SHERPA output. Uncertainties in emission inventories are known to be high, especially at the urban scale, as highlighted by Trombetti et al. (2018) who compared the features of different EU wide top-down inventories over major European urban areas. In this study, the impact of emissions from international shipping and from countries outside the model domain (boundary conditions) cannot be distinguished from each other and are, therefore, grouped under the category named “external”.

According to CityDelta modelling inter-comparison exercise (Cuvelier et al., 2007) concentrations and concentration changes are generally correlated (an underestimation of the concentration is likely to lead to an underestimation of the concentration change as well). For that reason, relative concentrations, expressed as concentration changes divided by concentration, are generally more robust than absolute values (concentration). To cope with this property of the model output and to facilitate comparison with those of other studies, the SHERPA results are expressed in terms of relative fractions of the PM_2.5_ reconstructed by the model. A more in depth discussion of the SHERPA methodology limitations is available in Thunis et al. (2018).

### 2.3 Study area

The study is focused on the main EU cities the Danube region, with the exception of Germany, and those of the WB (Table 1).

**Table 1:** List of the cities included in the present study. WB: Western Balkans, RDR: rest of the Danube region.

City	Country and code^a^	Region	City code	FUA population (x1000 inh.)	FUA area (km^2^)
Durrës	Albania, AL	WB	Dur	202	501
Elbasan	Albania, AL	WB	Elb	127	1249
Tirana	Albania, AL	WB	Tir	858	1266
Banja Luka	Bosnia and Herzegovina, BA	WB	Ban	199	9697
Sarajevo	Bosnia and Herzegovina, BA	WB	Sar	438	1404
Pristina	Kosovo, KS^b^	WB	Pri	199	2167
Podgorica	Montenegro, ME	WB	Pod	186	1437
Skopje	North Macedonia, MK	WB	Sko	467	1819
Belgrade	Serbia, RS	WB	Bel	1659	4065
Kragujevac	Serbia, RS	WB	Kra	179	2386
Niš	Serbia, RS	WB	Nis	260	2756
Novi-Sad	Serbia, RS	WB	Nov	342	4093
Subotica	Serbia, RS	WB	Sub	142	1676
Graz	Austria, AT	RDR	Gra	416	3703
Linz	Austria, AT	RDR	Lin	561	3522
Vienna/Wien	Austria, AT	RDR	Wie	2406	9205
Burgas	Bulgaria, BG	RDR	Bur	278	2948
Plovdiv	Bulgaria, BG	RDR	Plo	545	2773
Sofia	Bulgaria, BG	RDR	Sof	1543	5717
Varna	Bulgaria, BG	RDR	Var	418	2039
Brno	Czechia, CZ	RDR	Brn	789	3299
Liberec	Czechia, CZ	RDR	Lib	262	1327
Ostrava	Czechia, CZ	RDR	Ost	1123	3878
Pilsen/Plzen	Czechia, CZ	RDR	Plz	374	3103
Prague/Praha	Czechia, CZ	RDR	Pra	2205	6980
Ústí nad Labem	Czechia, CZ	RDR	Ust	248	874
Zagreb	Croatia, HR	RDR	Zag	1221	5059
Budapest	Hungary, HU	RDR	Bud	2928	6393
Bucharest/ Bucuresti	Romania, RO	RDR	Buc	2403	1066
Iasi	Romania, RO	RDR	Ias	465	799
Ljubljana	Slovenia, SI	RDR	Lju	546	2556
Bratislava	Slovakia, SK	RDR	Bra	618	2052
Košice	Slovakia, SK	RDR	Kos	362	1777

a ISO 3166-1 alpha-2 – two-letter country codes (ISO, 2013).

b This designation is without prejudice to positions on status, and is in line with UNSCR 1244 and the ICJ Opinion on the Kosovo declaration of independence.

Three countries (BA, RS and ME) belong to both the WB and the Danube region. For the data analysis, the six WB countries are pooled under a single group and the remaining Danube region counties are classified as “rest of the Danube region” (RDR). The 33 cities of the study have populations ranging from 0.12 to 2.95 million inhabitants and areas from 0.5 to 9.6 thousand km^2^ (Table 1).

## 3 Results

### 3.1 PM_2.5_ levels

**Fig. 2:**
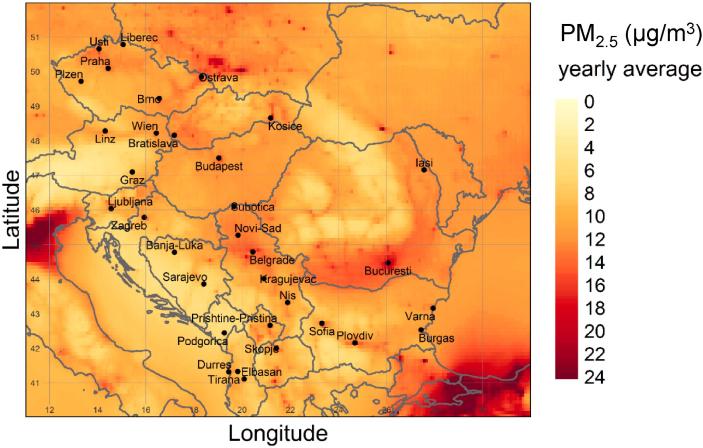
Modelled PM_2.5_ annual average (2014) concentrations over the study area.

The PM_2.5_ annual average concentration field generated over the study area is presented in Fig. 2. The map includes the countries of the Balkan Peninsula and neighbouring areas to appreciate polluted zones next to the study area that are possible sources of long-range transport of pollutants.

The highest PM_2.5_ levels in the regions surrounding the study area are those observed in the Po Valley, southern Poland and the Marmara region (Istanbul). Extended hot spots are also present within the study area, in southern Romania and northern Serbia while local urban hot spots correspond to the main cities.

In Fig. 1 the annual levels of PM_2.5_ simulated with the EMEP model for 2014 in the cities included in the present study are compared, wherever it is possible, with the corresponding measurements of this pollutant deriving from the official monitoring stations. Unfortunately, the PM_2.5_ measurements in urban background sites in the WB for the studied period are patchy and in the AIRBASE/EEA AQ database (https://www.eea.europa.eu/data-and-maps/dashboards/air-quality-statistics) PM_2.5_ annual averages from this area are available only since 2015. The modelled values are always lower than the measured ones. In the cities of the RDR EU Member States the differences range between 10% and 50%. The highest differences are observed in cities located near hot spots like Ostrava (CZ), Plovdiv (BG) and Sofia (BG). For the WB area the only available PM_2.5_ data for the cities included in this study are from RS (Belgrade and Novi Sad) and BA (Banja Luka) where the measured annual average is between 17% and 52% higher than the modelled one. PM_2.5_ measurements in other WB locations such as Pristina and small cities not covered in this study (Korca, AL; Shkoder, AL; Gorazde, BA; Prjiedor, BA and Karpos, MK) are available only for 2015 and 2016 (Fig. S3 of the Supplementary material). In these small settlements, the observed PM_2.5_ values are always the highest when compared with those for the same time window in Belgrade and the main cities in the RDR suggesting that local sources in small urban areas could have very high impacts.

Underestimation of the measured PM by air quality models is often reported in the scientific literature (e.g. Nopmongcol et al., 2012; Shimadera et al., 2016; Solazzo et al., 2017) and, as explained in section 2.2, can be attributed to various factors such as errors in the emission inventories, meteorology and chemistry of the secondary species. Nevertheless, air quality models have the advantage of providing consistent estimations for areas where little or no information is available (like the WB) with a uniform spatial resolution (usually gridded output) and full annual coverage. In addition, modelling is needed to estimate the impact of sources on pollution, and to accomplish scenario analysis to support the development of abatement strategies.

### 3.2 PM_2.5_ activity and spatial sources

In Table 2 are shown the overall impacts of the spatial sources for the WB and RDR areas. The transboundary (international) pollution is, on average, the main spatial source of PM_2.5_ in the studied area (44%; *p* < 0.0001) followed by FUA (22%) and national emissions (15%). The external (not apportioned) fraction represents 19% of the PM_2.5_. The Mann-Whitney non parametric test (which makes no a-priori assumption about the probability distribution of the variables) is used to assess whether there is a significant difference in sources' impacts between the two studied regions (StatTools v. 7.5). The FUA and transboundary contributions are on average slightly lower in the WB region compared with the RDR while the opposite is true for the national and external sources. However, as shown in Table 2, the differences between the two regions are not significant at the 1% level of significance (*p* < 0.01).

**Table 2:** Average of the PM_2.5_ shares (%) allocated to the geographical sources in all the cities, those in the Western Balkans (WB) and those in the rest of the Danube region (RDR). The Mann-Whitney tests between WB and RDR are also reported.

Region	FUA	International	National	Total apportioned	External
ALL average	22	44	15	81	19
WB average	19	40	16	76	24
RDR average	24	46	14	84	16
Mann-Whitney *p* value	0.1357	0.1671	0.4283	0.0193	0.0193

The city emissions impact between 30% and 40% of PM_2.5_ in the larger and most populated cities of the study area (Vienna, Sofia, Prague, Zagreb, Budapest, Bucharest) and also in some of the small ones (Linz, Bratislava). Not surprisingly, the cities contributing <10% to their own PM_2.5_ pollution are the smallest and less populated ones: Durrës, Elbasan, Sarajevo, Subotica, Burgas, Liberec and Ústí nad Labem. Despite the presence of outliers (Pristina and Ljubljana have low population and high FUA impact on PM_2.5_) the regression between the city population and the FUA impact on PM_2.5_ is statistically significant (R^2^ 0.3, p < 0.01) (Fig. S4 of the Supplementary material).

**Fig. 3:**
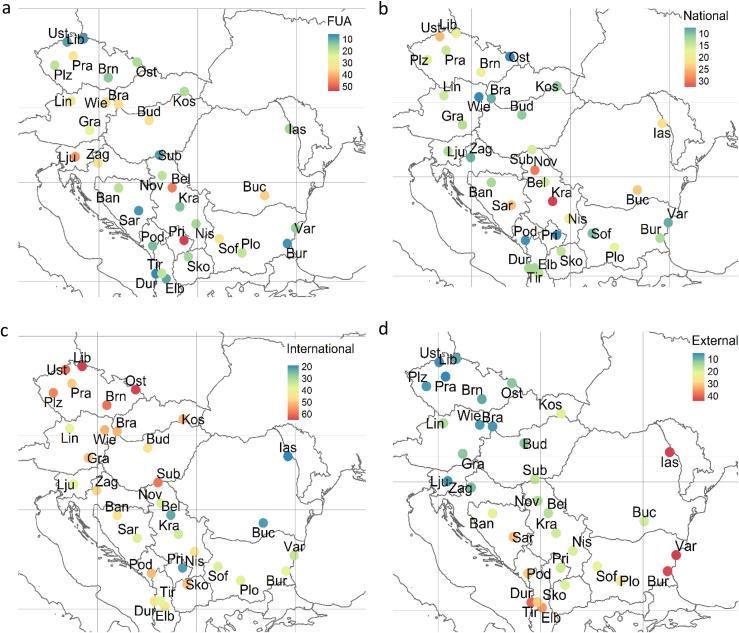
Percentage impact of geographical areas to the 33 studied cities estimated with SHERPA. FUA: functional urban area, national, international (within the model domain) and external (areas beyond the domain and sources not considered in this simulation e.g. international maritime). The values are provided in numeric format in Tables S2 and S3.

In Fig. 3 the percentage of PM_2.5_ originating from different areas, namely FUA (4-a), national (4-b), international (4-c) and external (4-d) are mapped. The data are also provided in numeric format (relative and absolute values) in Tables S2 and S3 of the Supplementary material. The influence of national emissions on the studied cities is the highest in the central part of the studied area (Fig. 3-b). It is between 20% and 30% in two of the studied Serbian cities (Kragujevac and Novi Sad), Sarajevo, Ústí nad Labem and the two Romanian cities (Bucharest and Iasi). On the contrary, low influences (<5%) are observed in Pristina and, Podgorica, both belonging to small countries and in Vienna and Ostrava, that are subject to higher than average transboundary contributions (see next paragraph).

The transboundary (= international) PM_2.5_ pollution is the highest in Czech and Slovakian cities followed by Vienna and Subotica. In Fig. 3-c, the clear NW-SE gradient of transboundary pollution suggest a possible influence from a geographical area located to the N or NW of the study area.

The external fraction encompasses the influence of sources outside the model domain and those from sources not allocated to a specific source. In Fig. 3-d the highest values are observed in two Bulgarian cities (Sofia and Varna), the Albanian cities and Iasi. With the exception of Sofia and Iasi, such high values of this fraction may be attributed to the influence of international maritime emissions respectively from the Black Sea/Marmara region and Adriatic Sea that are not allocated to any of the activity sectors nor geographical areas. Another explanation is the influence of sources located outside the domain (incorporated via the boundary conditions). A source apportionment study using receptor models and trajectory analyses reported non negligible contributions in Sofia of desert dust from the Saharan area in Northern Africa (mainly Libya and Algeria) and the area of the Caspian Sea corresponding to the North-western corner of the Turkestan desert (Perrone et al., 2018). Therefore, the high external impact observed in the abovementioned cities is also compatible with long range transport of mineral dust.

As shown in Table 3, the activity sources influencing most the PM_2.5_ levels in the cities of the entire study area are energy production, (22%), agriculture (19%) and residential combustion (16%). These three sources alone represent 57% of the total modelled PM_2.5_ and 71% of the apportioned PM_2.5_ (i.e. excluding the non-apportioned - external fraction, see Table 2). The extraction and distribution of fuels, use of solvents, other mobile machinery and waste are the least important PM_2.5_ sources. Their sum totalise only 6% of the total modelled PM_2.5_ and 7.5% of the apportioned PM_2.5_. According to the Mann-Whitney test, the relevance of some of the main sources changes significantly between the WB and the RDR. The test is significant (*p* < 0.01) for energy production, which is higher in the former, and for industrial combustion and agriculture, which are higher in the latter (Table 3). Also the mobile sources and industry (combustion and processes) are higher in the RDR, however, the differences are not significant at the 1% level of significance (Table 3). Although waste present significant differences among the two regions its impact on PM_2.5_ is not further discussed because of its limited importance (1–3%).

**Table 3:** Average (avg.) of the PM_2.5_ shares (%) allocated to the SNAP activity sectors in all the cities, those in the Western Balkans (WB) and those the rest of the Danube region (RDR). The Mann-Whitney tests between WB and RDR are also reported.

%	ENERGY	RESID COM	IND COM	IND PRO	EXTR FF	SOLV	TRA	OTH MOB	WASTE	AGRIC	Total apportioned
ALL avg.	22	16	4	5	<0.5	1	7	2	3	19	81
WB avg.	29	16	3	4	<0.5	1	6	1	1	14	76
RDR avg.	18	16	5	6	<0.5	1	8	3	3	23	84
Mann-Whitney *p* value	< 0.001	0.6189	< 0.0001	0.0094	–	0.5932	0.0374	0.0284	< 0.001	0.0007	0.0193

ENERGY: Energy production; RESID COM: residential combustion; IND COM: industrial combustion: IND PRO: industrial processes; EXTR FF: extraction and distribution of fuels; SOLV: use of solvents; TRA: road transport; OTH MOB: other mobile machinery; WASTE: waste treatment and disposal; AGRIC: agriculture.

**Fig. 4:**
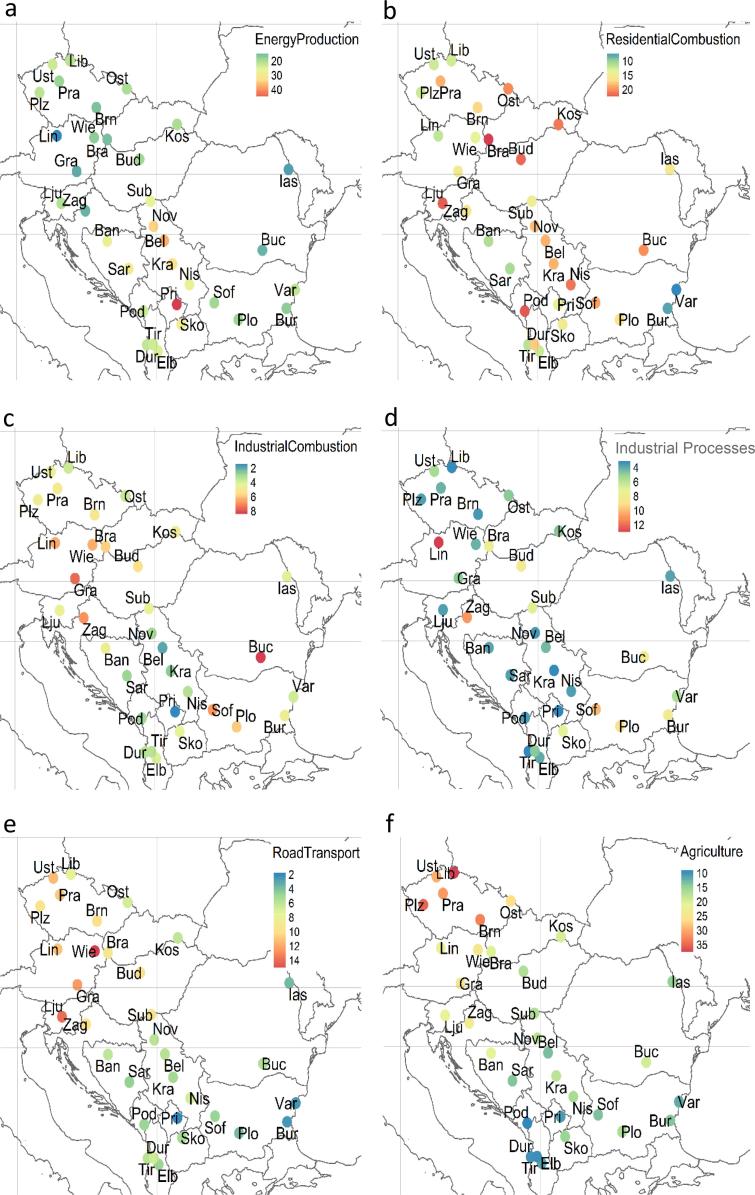
Percentage impact of selected activity sources to the 33 studied cities estimated with SHERPA. Energy production, Residential and commercial combustion, Combustion in Industry, Industrial (production) processes, Road transport and Agriculture. The values are provided in numeric format in Tables S2 and S3.

In Fig. 4 the relative impact of the most important activity sources in the 33 cities of this study is displayed on a map. The highest relative impacts of the energy sector are observed in the central part of the study area (RS, KS and BA) (Fig. 4-a). In the period 2010–2015, coal represented in these three countries between 50% and 58% of the total mix of fuels for energy supply expressed as kton/y (IEA, 2018). The impact of this source reaches almost 50% of the PM_2.5_ in Pristina followed by three Serbian cities (Belgrade, Novi Sad and Kragujevac) and Sarajevo where it ranges from 30% to 40%.

The cities with the highest impact from residential combustion are Bratislava, Podgorica, Ljubljana, Kosice, Budapest and Niš, all above 20% (Fig. 4-b). On the contrary, residential combustion contributes the least (6%) in two Bulgarian cities of the study (Burgos and Varna). Industrial combustion is the highest in Bucharest, Graz, Sofia, Zagreb, Linz and Vienna (6%–8%) while the impact of industrial processes is most important at Linz, Sofia, Zagreb, Plovdiv, Budapest, Bucharest and Bratislava (7%–13%)(Fig. 4-c and -d).

The road transport impacts most in the northern part of the studied area including all the three Austrian cities of the study, Ljubljana, Budapest, Zagreb and many Czech cities like Prague, Pilsen and Brno with impacts ranging from 9% to 16%. Very little influence (<4%) is observed in the Bulgarian cities and Pristina (Fig. 4-e). Also the impact of agriculture presents a clear North-South gradient with the highest values recorded in the Czech (25% - 38%), and Austrian cities (22% - 25%) followed by Zagreb (23%), Ljubljana (21%), Banja Luka (21%), Bratislava (20%) and Kosice (20%) (Fig. 4-f). The lowest influence of this source is observed in the South-West part of the study area (Albania, Montenegro, Kosovo and Bulgaria) where the values are below 12%. The same pattern is observed in the absolute values (μg/m^3^) suggesting that it is not depending on the relative importance of this source compared to others. One possible explanation is the influence of long-range transport of aerosol from areas with high NH_3_ emissions such as Northern Italy, Switzerland and Southern Germany (TNO MACC III, Kuenen et al., 2014), as discussed below.

**Fig. 5:**
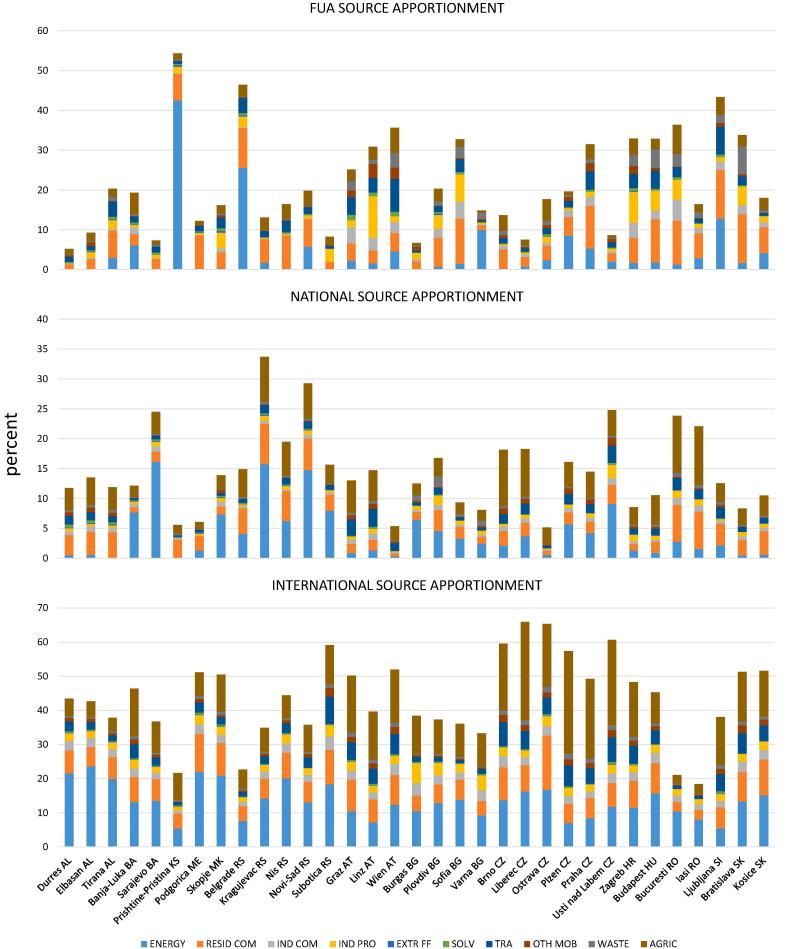
Bar plots of the impact of geographical sources on PM_2.5_ in the 33 studied cities split by activity sectors. ENERGY: Energy production; RESID COM: residential combustion; IND COM: industrial combustion: IND PRO: industrial processes; EXTR FF: extraction and distribution of fuels; SOLV: use of solvents; TRA: road transport; OTH MOB: other mobile machinery; WASTE: waste treatment and disposal; AGRIC: agriculture.

The combined analysis of geographical and activity sources shown in Fig. 5 (available also in numeric format in Table S4 of the Supplementary material) highlights the variable importance of the macrosectors not only between cities but also at different geographical scales.

**Fig. 6:**
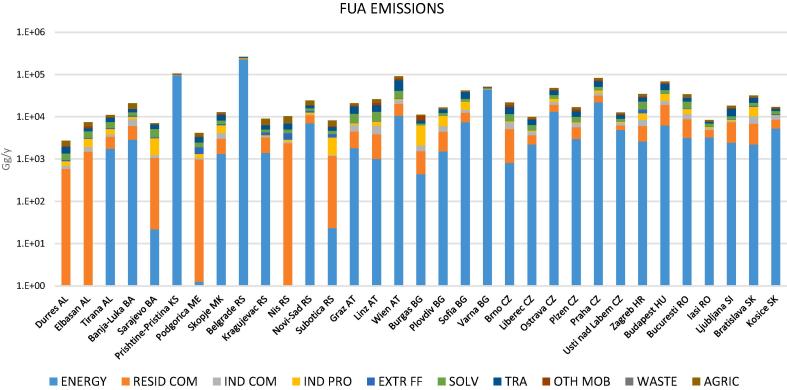
FUA emissions split by activity sectors used as input for the model to estimate the impact of sources on PM_2.5_ in the cities of this study. Same abbreviations as Fig. 5.

The energy sector is clearly the responsible of the high FUA contributions in Pristina, Belgrade and to a lesser extent in Ljubljana. In the first two cases, such a high impact is explained by the very high emissions of this source in these two cities, which are one order of magnitude higher than those of the average of the other cities (Fig. 6 and Fig. S5). In Fig. 5, a more balanced blend of sources is observed for the other cities with FUA impacts above 30% like Prague, Bucharest, Budapest, Zagreb, Vienna and Linz. In these cities, also residential heating and industry are contributing to PM_2.5_ considerably.

In the PM_2.5_ fraction allocated to national emissions outside the studied cities (Fig. 5) the influence of the energy sector and agriculture becomes clearer and, due to the homogeneity of the model input data (Fig. S5), there is higher similarity between the cities of the same country. The cases where energy has a very low impact are Pristina, Zagreb, Budapest and the Albanian, Austrian and Slovakian cities (Fig. 5).

When it comes to the transboundary (= international) emissions, the split of the input emission data among activity sources is almost the same for all the cities (sum of all emissions outside each country, Fig. S5) and, therefore, the difference in the source impacts (Fig. 5) are mostly explained by atmospheric processes like advection and ageing. Consequently, at the international scale there is a higher uniformity in the source impacts among cities from different countries than at the local scale, where the local emission mix is more heterogeneous and has a dominant effect. In addition, the high impact of sources like agriculture, energy and, to a lower extent, road transport, which emit PM_2.5_ gaseous precursors, is coherent with the long-range transport and associated chemical transformations leading to the formation of secondary aerosol. Moreover, the share of the residential sector in the transboundary fraction is higher in the majority of the cities located in the NW quadrant of the study area and, therefore, are likely originated in an area located to the N or NW of the study area.

The split of activity sources by their geographical origin is summarised in Fig. S6. The combined analysis of activity and geographical sources leads to the conclusion that the share of PM_2.5_ allocated to transboundary is the highest for Energy production (*p* < 0.0001) and Agriculture (p < 0.0001) while the residential combustion impact on PM_2.5_ is mainly deriving from both transboundary and from the same FUA (p < 0.0001). For the other sources, the between-city variability of their impacts on PM_2.5_ is higher (in particular at the FUA and national levels) and therefore the spatial patterns are not significant.

### 3.3 Biomass burning in the residential sector

**Fig. 7:**
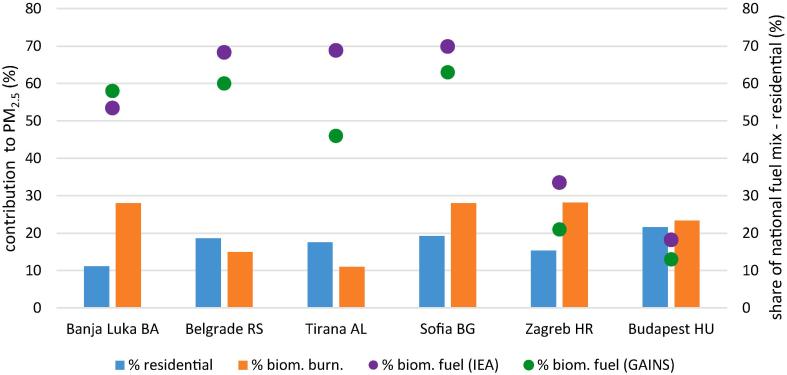
Impacts of the residential sector to PM_2.5_ estimated with SHERPA (this study, blue bars) and biomass burning contributions obtained with receptor models (from the literature, orange bars) in six cities of the study. The shares of biomass burning to the national fuel mix in the residential sector reported by IEA for 2012 (purple dots) and in the GAINS CLE scenario for 2013 (green dots) are also displayed for the respective countries. (For interpretation of the references to color in this figure legend, the reader is referred to the web version of this article.)

In the previous sections the impact of the residential sector on PM_2.5_ in the studied cities was analysed in detail with a breakdown by geographical areas. However, the emission inventory used for the study does not provide fuel details for domestic heating. In order to gain an insight into the specific role of biomass burning, the contributions obtained with receptor models (RMs) from the literature (Almeida et al., 2019; Perrone et al., 2018) between 2013 and 2015 for six of the studied cities (Banja Luka, Belgrade, Tirana, Sofia, Zagreb and Budapest) are compared with the SHERPA residential sector impacts (Fig. 7). For the interpretation of these results, it should be taken into account that the RMs estimate the mass transferred from the source to the outdoor concentration while the approach adopted in SHERPA (brute force or emission reduction impact) estimates the changes in concentrations deriving from changes in emissions. The results of these two approaches differ for pollutants with non-linear behaviour (e.g. secondary inorganic aerosol). In addition, it is worth mentioning that receptor models do not apportion the secondary inorganic aerosol to its sources. Therefore, the secondary sulphate, nitrate and ammonium that in the SHERPA results are allocated to the sources of their gaseous precursors: sulphur dioxide (e.g. energy production), nitrogen oxides (e.g. road traffic) and ammonia (e.g. agriculture), in RMs results are pooled under the category “secondary inorganic aerosol”. Such effect, however, affects the biomass burning only to a limited extent because its contribution to the formation of ammonium nitrate and ammonium sulphate is very limited; on average only 6% of the mass allocated to fresh (not aged) biomass burning corresponds to these compounds (SPECIEUROPE; Pernigotti et al., 2016). For sources contributing substantially to the secondary inorganic aerosol, the comparison between RMs and CTM brute force approach is not straightforward (Belis et al., 2019).

As already observed in Fig. 1, the measured PM_2.5_ of the RM studies, covering the full year (i.e. the cold and warm seasons), are in general higher than the modelled PM_2.5_. For that reason, in this section only the relative contributions are discussed with a view to understand the role of biomass burning within the residential impacts identified with SHERPA. According to the abovementioned receptor model studies, the relative contributions of the biomass burning to PM_2.5_ range between 10% in Tirana and 28% in Banja Luka, Sofia and Zagreb while those of SHERPA (this study) for residential combustion range between 11% in Banja Luka and 22% in Budapest. A closer look shows that in Budapest the two estimations are comparable, biomass burning relative contributions are lower than those of the residential sector in Tirana and Belgrade while the contrary is true for Banja Luka, Zagreb and Sofia (Fig. 7). These results lead to the conclusion that in the former three cities biomass burning is the dominant source within the residential sector while in the latter three ones the lower modelled impact of the residential sector compared to the sole biomass burning suggests that role of this sector is underestimated by the model used in this study (the same relationships are observed when analysing the absolute values).

Another indication of the relevance of biomass burning in the residential sector derives from the fuel mix in this sector at the country level, which is the basis for the estimation of the biomass burning emissions (IEA, 2014; IEA, 2016). Muntean et al. (2017) analysed the evolution of the different fuels' shares in the residential sector from 1990 to 2012. High biomass fuel shares (> 50%) with a relatively stable trend are observed in AL, BA, RO, RS and ME. In SI and BG, an increase in the use of biomass in the residential sector took place at the end of the nineties at the expense of coal and liquid fuels. A progressive increase is also observed in CZ along the entire period matching a decrease in the use of coal. The percentages of biomass fuel consumption in the residential sector are relatively low and stable in HR (≈ 30%), HU (< 20%) and SK (< 10%). Similar estimations about the biomass fuel share in the domestic sector from the GAINS scenario PRIMES 2013 REF-CLE (IIASA, 2014) are between 3% and 23% points lower than those of the IEA for 2012, with the exception of BA which is 4% points higher in GAINS.

In Fig. 7, the IEA country shares of biomass fuels in the residential sector (in energy units) for 2012 (Muntean et al., 2017) and those of GAINS (IIASA, 2014) for 2013 are compared with the contributions from biomass burning estimated by RMs and the impact of residential sector by SHERPA at the city level (this study). The highest shares of biomass in the fuel mix are observed in AL, BA, BG and RS (40% - 70%) while those of HR and HU are relatively low (10–40%). A number of European studies comparing the use of biomass for residential combustion in small settlements and in large cities have shown that their share is higher in the former ones (Braniš and Domasová, 2003; Caseiro et al., 2009; Larsen et al., 2012; Piazzalunga et al., 2011). Therefore, the share of biomass in the mix of fuels of large cities is expected to be lower than the national average. In line with this hypothesis, the national share of this fuel in the domestic sector is higher than the contributions of biomass burning to PM_2.5_ in most cities (Banja Luka, Belgrade, Tirana and Sofia; Fig. 7). On the contrary, the contributions of biomass burning to PM_2.5_ at the city level are comparable to the share of this fuel in the residential sector at the national level in Zagreb and Budapest. In these locations, the national shares of biomass fuel are well below the average for the Danube and WB regions (40%) suggesting that either the emissions are underestimated or the biomass is not commonly used as fuel in households in these countries. The higher contributions (35%) of biomass burning observed in a small Hungarian city not included in the present study (Debrecen; Almeida et al., 2019) compared to those shown for Budapest in Fig. 7, depose in favour of the first hypothesis.

## 4 Discussion

### 4.1 Geographical sources

The SHERPA air quality screening tool provided new evidence on the importance of transboundary PM pollution (average 44%, std. dev. 12%) in the studied area. The impact of this source in the Danube region is 24% points (*p* < 0.0001) higher than in the cities from the EU28 excluding those in this region (hereafter referred to as “rest of Europe”, RE) resulting from the dataset used for the study by Thunis et al. (2018). The high relative impact of this geographical source is in line with previous work on the sources of PM in this area (Nicolae et al., 2008; Perrone et al., 2018). A PM_2.5_ hot spot in Poland is clearly visible in Fig. 1 and in the report on air quality in Europe (EEA, 2016; map 4.1 p. 28). The abovementioned study with SHERPA tool (based on CHIMERE model) has shown that in Poland domestic heating is the predominant source associated with local emissions (Thunis et al., 2018). According to Fig. 5, most of the cities located in the north-western quadrant of the study area (Ostrava, Kosice, Subotica, Brno, Graz, Budapest, Vienna, Bratislava, Liberec and Zagreb), are those with the highest share of domestic heating in the PM_2.5_ fraction associated with transnational pollution. The proximity to the Polish border suggests a transboundary impact of the domestic combustion in Poland on these cities.

The international maritime traffic are suspected to contribute most in the areas next to the Adriatic Sea and Black Sea/Marmara region. However, since this source is included in the external category the influence of emissions outside the model domain cannot be excluded.

### 4.2 Sectorial sources

The activity sources influencing most the PM_2.5_ levels in the cities of the study area are energy production, (22%) agriculture (19%), residential combustion (16%) and road transport (7%).

As shown in Table 3, in the WB cities the impact of energy production on PM_2.5_ is significantly higher (*p* < 0.0001) than in RDR (29% and 18%, respectively). Moreover, according to the abovementioned dataset by Thunis et al. (2018), the impact of this source in the RDR cities is significantly higher (p < 0.0001) than in the RE ones (18% and 9%, respectively). The considerable impact of the energy production in the WB compared to both the RDR and RE has been associated with the high share of coal-fuelled power plants in this region. Between 2010 and 2015, coal represented 50% of the total mix of fuels used for energy purposes in the WB region (IEA, 2018). The high share of coal in the energy mix of Serbia and Kosovo (>50%, see section 3.1) also explains the very high contribution of the energy sector to PM_2.5_ in Belgrade and Pristina (Fig. 5). According to the GAINS scenario PRIMES 2013 REF-CLE (IIASA, 2014) the great majority of the emissions from the energy sector in the WB (>95%) is produced by coal burning while in the RDR the share is <80%. In addition, this scenario suggests that the PM_2.5_ emission efficiency of coal power plants (53 t of PM_2.5_ per PJ of energy produced) in the WB is one order of magnitude lower than the one of those in the RDR (3 t of PM_2.5_/PJ).

The impact of agriculture on PM_2.5_ is significantly higher (*p* < 0.0005) in the RDR cities than in the WB ones (23% and 14% respectively) while the impact of this source in the former region is comparable with the ones in the RE. The opposite situation is observed in road transport which shows comparable impacts on PM_2.5_ in WB and RDR cities (6% and 8%, respectively) while the latter is significantly lower (p < 0.0001) than in the RE cities (8% and 15% respectively). The road transport and agriculture show noticeable spatial gradient with higher impacts in the northern part of the studied area. Even though it cannot be excluded that the EMEP model spatial resolution and VOC scheme could have led to the underestimation of this source the fact that the problem arises only in the WB region suggests that it is most likely due to the emission input data than to deficiencies in the model.

The SHERPA tool shows that emissions from the residential sector account for a share between 7% and 24% of PM_2.5_ in the 33 studied cities. The city with the highest impact from residential combustion (> 20%) are Bratislava, Podgorica, Ljubljana, Kosice, Budapest and Niš.

No significant difference is observed in the impact of residential combustion between the WB and RDR cities (16% in both) and between RDR and RE cities (16% and 12%, respectively). However, the comparison with receptor models suggest that the role attributed to this source by the CTMs is likely underestimated, at least in some cities (Banja Luka, Zagreb and Sofia). Such differences have been associated with the considerable uncertainties about the biomass combustion emission factors and the actual consumption (activity data) of this fuel in households (Monforti-Ferrario and Belis, 2018; Piazzalunga et al., 2011).

An investigation on the data of fuel consumption, which are the basis for the development of emission inventories, indicate that figures can vary considerably among countries and among authors. In 2012, the share of biomass use in the residential sector in 12 countries of the RDR and WB regions was on average 48% (std. dev 24%) (Muntean et al., 2017). Also the PRIMES 2013 REF-CLE (IIASA, 2014) indicates a high share of the emissions from the residential sector in the Danube region derive from biomass burning. Such variable but relatively high share of biomass fuel in the residential sector at the country level suggests a higher role of this source in the country side and small settlements than the one predicted in this study by the CTM for the main cities. This is not the case in some countries (HU, HR) where the share of biomass in the national residential combustion fuel mix is relatively low (<40%). At least in one of these countries there are indications of high contributions of biomass burning in small settlements. One explanation could be the use of self-produced biomass fuel, which is not recorded in the official fuel registers, to cause the underestimation of the actual emissions.

Another source of uncertainty are the limitations of the modelling approaches. The EMEP VOC scheme could have led to an underestimation of the carbonaceous organic fraction emitted by this source and consequently the sources where this fraction is relevant such as residential combustion and road transport. This would be in line with the model performance evaluation that showed a considerable underestimation of this fraction in PM_2.5_ (section 2.2). Also the uncertainties in the receptor model estimations may have contributed to the abovementioned differences with the CTM derived estimations. The contribution from long-range transport of open fires erroneously allocated by RMs to biomass burning in households may lead to an overestimation of the role of this source in cities. However, it has been shown that RMs are able to efficiently split local (fresh) and long range transport (aged) biomass burning sources thanks to the presence of markers for secondary aerosol (e.g. ammonium nitrate and sulphate) and the different temporal patterns in the latter (Gunsch et al., 2018; Perrone et al., 2018).

### 4.3 Combined use of geographical and sectorial sources

The combined analysis of sectorial and spatial sources provided by SHERPA provides evidence to bring air quality management into focus. Agriculture and Energy are the sources most associated with the transboundary pollution (international source). It has also been observed that residential combustion is in part associated to international geographic sources (Fig. S6). As shown in Fig. 5, the highest share of the residential combustion in the international PM_2.5_ fraction is observed in the north western part of the studied area. A possible advection of pollutants from Poland has been hypothesised in section 4.1. Considering that in this country coal is the main fuel used in the residential sector (46%, PRIMES 2013 REF-CLE; IIASA, 2014) and biomass burning represents only 17%, the residential sector associated with transboundary pollution is likely deriving mainly from coal combustion. On the contrary, the residential combustion originated within the cities of the WB and RDR regions is mainly associated with biomass burning as discussed in section 3.3.

### 4.4 Policy implications

The information about the geographical origin of pollution is necessary to identify the level at which measures should be taken. According to the results of this study, measures are necessary at the international level for Energy and Agriculture sources, in line with the directive on large combustion plants, the national emissions ceilings and the EU common agricultural policy. In particular, the coal-fuelled power plants result as one of the key sectors to tackle in the WB. On the contrary, the measures concerning residential combustion are more appropriate at the local (municipal, regional) level taking advantage, for instance, of the air quality plans in the frame of the Air Quality Directive. In addition to mandatory legislation, important initiatives at the local levels have become of considerable importance in recent years such as the EU partnership on Air Quality and the Covenant of Majors. Actions involving most countries of the Balkan area may also be taken in the frame of the EU macro-regional policy which involves EU and non-EU countries (e.g. EU strategy for the Danube Region and EU strategy for the Adriatic and Ionian Region).

The legislation in the field of energy and climate of the EU and the non-EU countries of the studied regions promotes the use of biomass for energy purposes, including domestic heating. Considering the potential risks for the human health and the environment of biomass burning, mainly because of emissions of air pollutants and overexploitation of natural resources, control is necessary to use biomass fuel both safely and sustainably. A number of EU directives are currently available in the areas of eco-design, energy labelling of appliances and energy efficiency in buildings, to streamline the use of biomass for energy purposes. In addition, the green procurement, bio-economy and circular economy concepts can also be applied to orient policies for the appropriate use of the biomass resources.

## 5 Conclusions

The EMEP version of the SHERPA tool has been used in this study to obtain information about the sources of PM_2.5_ in Southern East Europe with particular reference to the Western Balkans where the still scarce available information indicate the levels of this pollutant are among the highest of Europe.

The results of the comparison between the modelled impact of sources and those deriving from measurements suggest the need to put more effort in reducing the uncertainties in the model input data (e.g. emissions from combustion of biomass in the residential sector and emissions from the energy sector in specific cities of the study area). Improvement of the models to achieve a more satisfactory reconstruction of the PM and therefore enhance the confidence on the model source apportionment outcome is also needed. In this regards, a better reconstruction of the thermal inversion during winter time and improvements in the mechanisms leading to the formation of SIA and SOA would contribute to obtain more reliable estimation of key diffuse emissions such as those from biomass burning and traffic. This study confirms that taking advantage of source information deriving from both CTM and receptor models leads to a better understanding of the source impacts.

## Appendix A Supplementary data

Supplementary material Image 1
