# Supplementary material for: Urban pollution in the Danube and Western Balkans regions: The impact of major PM2.5 sources
Source: Environ Int. 2019 Dec;133(Pt A):105158. doi: 10.1016/j.envint.2019.105158 (PMC6839612; doi:10.1016/j.envint.2019.105158)
Supplement: Supplementary material [file mmc1.pdf]

Urban pollution in the Danube and Western Balkans regions: the impact of major $PM_{2.5}$ sources

by C.A. Belis, E. Pisoni, B. Degraeuwe, E. Peduzzi, P. Thunis, F. Monforti-Ferrario, D. Guizzardi

Environment International, article number: 105158

Supplementary Material

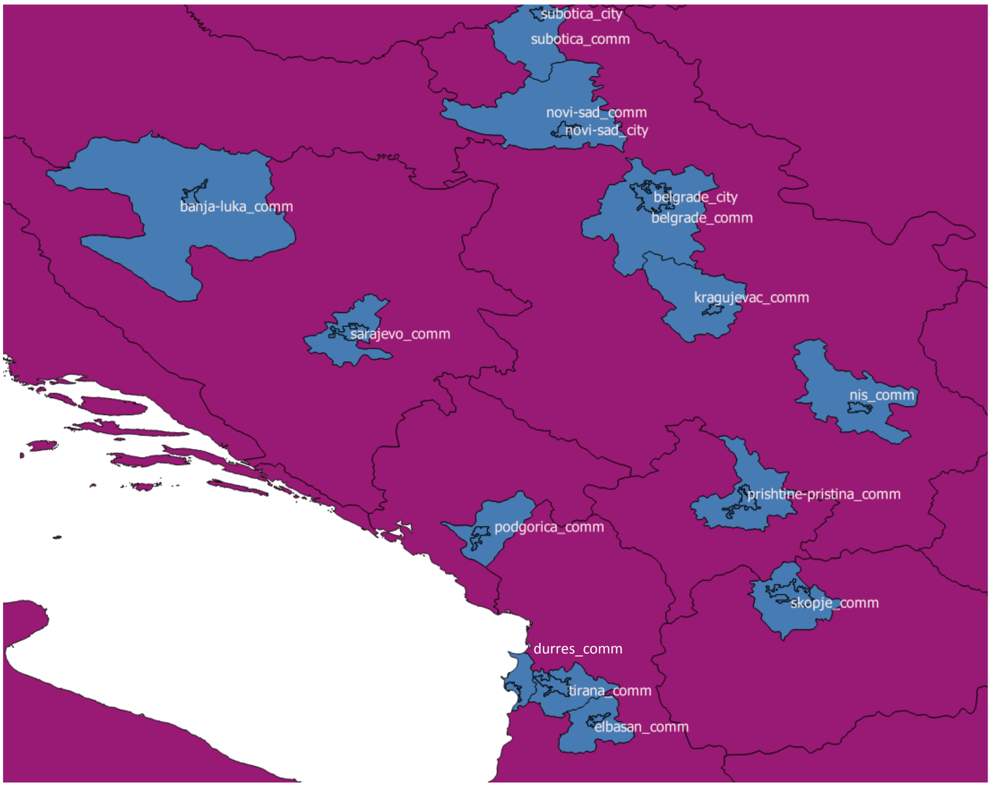


***Figure S1. Polygons of the Functional Urban Areas of the Western Balkan countries used in this study. The city cores are also shown even though they were not used for the calculations because too small when compared with the spatial resolution of the grid.***

***Table S1. Comparison of model results and observations for 2014. (EMEP, 2016)***

| Component | units | fraction | N stat. | Obs. | Mod. | Bias (%) | RMSE | Corr. | IOA |
|---|---|---|---|---|---|---|---|---|---|
| PM10 | (µg/m$^3$) | | 33 | 13.95 | 10.58 | -24 | 4.89 | 0.74 | 0.74 |
| PM2.5 | (µg/m$^3$) | | 27 | 9.47 | 8.26 | -13 | 2.92 | 0.77 | 0.84 |
| sulphate , incl sea salt | (µg/m$^3$) | TSP | 28 | 1.74 | 1.27 | -27 | 0.69 | 0.8 | 0.76 |
| sulphate , incl sea salt corrected | (µg/m$^3$) | TSP | 20 | 1.56 | 1.02 | -34 | 0.79 | 0.83 | 0.74 |
| sulphate | (µg/m$^3$) | $PM_{10}$ | 12 | 1.61 | 0.94 | -42 | 0.72 | 0.85 | 0.66 |
| sulphate | (µg/m$^3$) | $PM_{2.5}$ | 7 | 1.98 | 1.02 | -24 | 1.77 | 0.54 | 0.5 |
| NO−3 | (µg/m$^3$) | TSP | 15 | 1.14 | 1.26 | 11 | 0.46 | 0.81 | 0.89 |
| NO−3 | (µg/m$^3$) | $PM_{10}$ | 12 | 1.13 | 1.34 | 19 | 0.47 | 0.91 | 0.92 |
| NH+4 | (µg/m$^3$) | TSP | 19 | 0.82 | 0.79 | -4 | 0.23 | 0.87 | 0.93 |
| EC | (µgC/m$^3$) | $PM_{2.5}$ | 10 | 0.43 | 0.34 | -22 | 0.28 | 0.64 | 0.76 |
| OC | (µgC/m$^3$) | $PM_{2.5}$ | 10 | 2.37 | 1.07 | -55 | 1.38 | 0.9 | 0.53 |
| Na+ | (µg/m$^3$) | TSP | 22 | 0.69 | 0.73 | 5 | 0.62 | 0.71 | 0.83 |
| Na+ | (µg/m$^3$) | $PM_{10}$ | 6 | 0.69 | 0.2 | -71 | 0.86 | 0.93 | 0.45 |

N stat: number of stations, RMSE: root mean square error, Corr.: correlation coefficient, IOA: index of agreement.

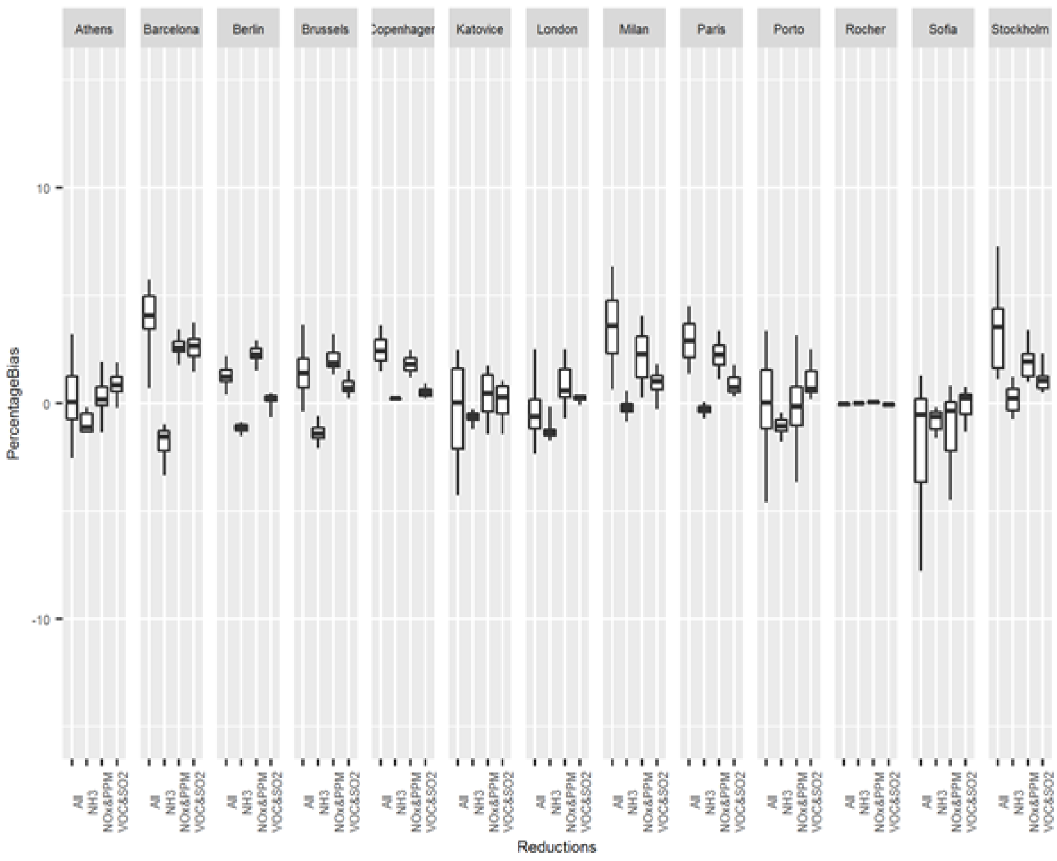


***Figure S2. Validation of SHERPA emission reduction scenario by comparison with the corresponding EMEP value for five precursors: NH3, NOX, primary PM, VOC and SO2 in 13 European cities.***

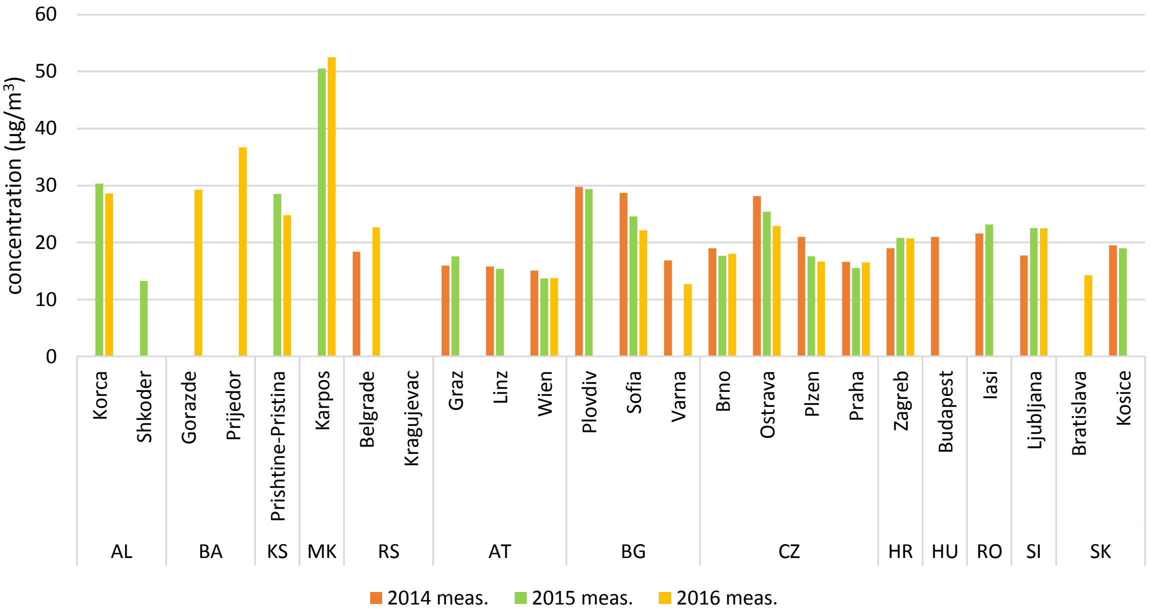


***Figure S3. $PM_{2.5}$ concentrations for some Danube and Western Balkans cities (source EEA and SEPA).***

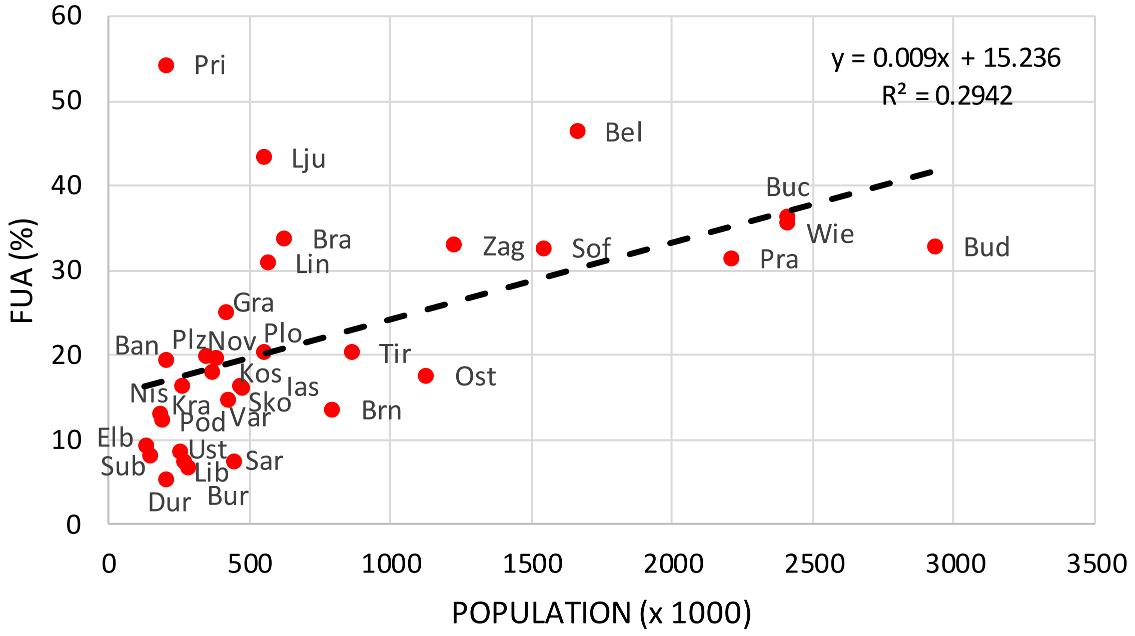


***Figure S4. Scatter plot showing the relationship between the city (Functional Urban Area) population and the impact of the city emissions to its own $PM_{2.5}$ levels, in the study area.***

***Table S2. Impacts (%) of the areas and SNAP macrosectors on the $PM_{2.5}$ concentrations of the 33 studied cities.***

| City name | City (FUA) | International | National | External | ENERGY | RESID. COMB. | IND. COMB. | IND. PROC. | EXTR. FOSSIL FUELS | SOLVENTS | TRAFFIC | OTH. MOBILE | WASTE | AGRICULTURE | Total |
|---|---|---|---|---|---|---|---|---|---|---|---|---|---|---|---|
| Durres AL | 5.2 | 43.4 | 11.8 | 39.6 | 22.1 | 11.5 | 3.4 | 3.0 | 0.4 | 1.1 | 5.8 | 2.0 | 1.1 | 10.2 | 60.4 |
| Elbasan AL | 9.3 | 42.7 | 13.5 | 34.5 | 24.1 | 12.3 | 3.9 | 3.9 | 0.5 | 1.0 | 4.8 | 2.6 | 1.1 | 11.3 | 65.5 |
| Tirana AL | 20.3 | 37.8 | 11.9 | 30.0 | 23.0 | 17.5 | 3.4 | 4.7 | 0.4 | 1.3 | 6.6 | 1.5 | 1.9 | 9.8 | 70.0 |
| Banja-Luka BA | 19.3 | 46.3 | 12.1 | 22.2 | 27.0 | 11.1 | 4.5 | 3.7 | 0.4 | 0.9 | 6.0 | 1.5 | 1.5 | 21.2 | 77.8 |
| Sarajevo BA | 7.3 | 36.7 | 24.5 | 31.4 | 29.8 | 10.7 | 2.8 | 3.6 | 0.4 | 0.9 | 4.5 | 0.8 | 1.3 | 13.9 | 68.6 |
| Prishtine-Pristina KS | 54.3 | 21.6 | 5.6 | 18.5 | 47.9 | 14.1 | 1.3 | 2.9 | 0.6 | 0.7 | 1.8 | 0.3 | 1.0 | 10.8 | 81.5 |
| Podgorica ME | 12.3 | 51.1 | 6.1 | 30.5 | 23.2 | 22.4 | 2.9 | 3.5 | 0.6 | 0.8 | 4.8 | 1.2 | 1.0 | 9.1 | 69.5 |
| Skopje MK | 16.2 | 50.5 | 13.9 | 19.4 | 28.6 | 15.0 | 4.2 | 6.9 | 0.8 | 1.3 | 5.2 | 1.7 | 1.5 | 15.3 | 80.6 |
| Belgrade RS | 46.4 | 22.7 | 14.9 | 16.0 | 37.3 | 18.6 | 1.9 | 4.3 | 0.5 | 0.9 | 5.7 | 0.8 | 0.9 | 13.2 | 84.0 |
| Kragujevac RS | 13.1 | 34.8 | 33.7 | 18.4 | 31.6 | 18.6 | 2.6 | 3.0 | 0.4 | 0.6 | 5.1 | 0.9 | 1.2 | 17.6 | 81.6 |
| Nis RS | 16.5 | 44.4 | 19.5 | 19.6 | 26.4 | 21.1 | 3.2 | 3.5 | 0.3 | 0.6 | 6.8 | 1.1 | 1.2 | 16.1 | 80.4 |
| Novi-Sad RS | 19.8 | 35.8 | 29.3 | 15.1 | 33.6 | 18.3 | 2.9 | 3.4 | 0.2 | 0.6 | 5.9 | 1.3 | 1.2 | 17.6 | 84.9 |
| Subotica RS | 8.3 | 59.1 | 15.7 | 17.0 | 26.3 | 14.7 | 4.3 | 6.8 | 0.2 | 0.4 | 9.6 | 2.9 | 1.2 | 16.7 | 83.0 |
| Graz AT | 25.2 | 50.2 | 13.0 | 11.6 | 13.5 | 15.2 | 7.5 | 4.8 | 0.1 | 1.9 | 12.4 | 4.0 | 4.1 | 24.8 | 88.4 |
| Linz AT | 30.9 | 39.7 | 14.7 | 14.7 | 10.1 | 11.7 | 6.3 | 13.2 | 0.1 | 1.8 | 11.1 | 5.9 | 2.7 | 22.4 | 85.3 |
| Wien AT | 35.6 | 51.9 | 5.4 | 7.0 | 17.2 | 13.9 | 6.3 | 4.1 | 0.1 | 1.4 | 15.7 | 5.2 | 5.0 | 24.3 | 93.0 |
| Burgas BG | 6.8 | 38.4 | 12.5 | 42.3 | 17.1 | 7.9 | 4.5 | 8.0 | 0.1 | 0.5 | 2.5 | 1.6 | 1.6 | 13.9 | 57.7 |
| Plovdiv BG | 20.4 | 37.3 | 16.8 | 25.6 | 18.1 | 16.3 | 5.8 | 8.7 | 0.5 | 0.8 | 3.8 | 0.7 | 3.4 | 16.3 | 74.4 |
| Sofia BG | 32.7 | 36.1 | 9.4 | 21.8 | 18.6 | 19.3 | 6.6 | 10.1 | 0.2 | 0.8 | 4.7 | 0.7 | 3.9 | 13.4 | 78.2 |
| Varna BG | 14.8 | 33.3 | 8.1 | 43.8 | 21.7 | 6.6 | 3.7 | 5.5 | 0.1 | 0.5 | 2.4 | 1.0 | 2.9 | 11.9 | 56.2 |
| Brno CZ | 13.7 | 59.5 | 18.2 | 8.6 | 16.1 | 16.9 | 4.9 | 3.4 | 0.1 | 0.5 | 10.1 | 3.7 | 2.8 | 32.9 | 91.4 |
| Liberec CZ | 7.5 | 65.9 | 18.3 | 8.2 | 20.7 | 12.5 | 3.6 | 3.0 | 0.1 | 0.7 | 7.9 | 2.8 | 2.3 | 38.1 | 91.8 |
| Ostrava CZ | 17.7 | 65.4 | 5.2 | 11.8 | 19.7 | 20.3 | 3.6 | 4.7 | 0.3 | 1.0 | 6.8 | 2.5 | 2.9 | 26.5 | 88.2 |
| Plzen CZ | 19.6 | 57.4 | 16.1 | 6.9 | 21.2 | 12.4 | 4.5 | 3.6 | 0.1 | 0.7 | 9.5 | 3.6 | 2.3 | 35.1 | 93.1 |
| Praha CZ | 31.5 | 49.2 | 14.5 | 4.8 | 18.0 | 18.5 | 4.9 | 4.0 | 0.1 | 0.7 | 10.9 | 4.2 | 2.5 | 31.4 | 95.2 |
| Usti nad Labem CZ | 8.7 | 60.7 | 24.8 | 5.8 | 22.9 | 12.3 | 4.5 | 5.4 | 0.2 | 1.3 | 11.3 | 4.4 | 2.0 | 29.9 | 94.2 |
| Zagreb HR | 33.0 | 48.3 | 8.6 | 10.2 | 14.5 | 15.3 | 6.8 | 10.9 | 0.3 | 1.1 | 9.9 | 3.8 | 4.3 | 23.0 | 89.8 |
| Budapest HU | 32.9 | 45.3 | 10.5 | 11.3 | 18.5 | 21.6 | 5.4 | 8.2 | 0.1 | 0.6 | 9.7 | 2.3 | 5.7 | 16.7 | 88.7 |
| Bucuresti RO | 36.4 | 21.1 | 23.9 | 18.7 | 14.4 | 20.0 | 8.3 | 8.1 | 0.1 | 0.7 | 5.5 | 0.7 | 4.2 | 19.4 | 81.3 |
| Iasi RO | 16.4 | 18.4 | 22.1 | 43.1 | 12.6 | 15.3 | 4.0 | 3.6 | 0.0 | 0.2 | 3.6 | 0.7 | 2.2 | 14.7 | 56.9 |
| Ljubljana SI | 43.3 | 38.0 | 12.5 | 6.1 | 20.3 | 22.2 | 4.4 | 3.5 | 0.2 | 1.3 | 13.9 | 3.0 | 3.6 | 21.5 | 93.9 |
| Bratislava SK | 33.8 | 51.3 | 8.3 | 6.6 | 15.5 | 23.5 | 5.7 | 7.8 | 0.1 | 0.4 | 9.1 | 2.7 | 8.4 | 20.1 | 93.4 |
| Kosice SK | 18.0 | 51.6 | 10.5 | 19.9 | 19.9 | 20.9 | 4.7 | 4.5 | 0.1 | 0.3 | 6.1 | 1.8 | 1.9 | 19.8 | 80.1 |

***Table S3. Impacts (µg/m³) of the areas and SNAP macrosectors on the $PM_{2.5}$ concentrations of the 33 studied cities.***

| City name | City (FUA) | International | National | External | ENERGY | RESID. COMB. | IND. COMB. | IND. PROC. | EXTR. FOSSIL FUELS | SOLVENTS | TRAFFIC | OTH. MOBILE | WASTE | AGRICULTURE | Total |
|---|---|---|---|---|---|---|---|---|---|---|---|---|---|---|---|
| Durres AL | 0.5 | 4.3 | 1.2 | 3.9 | 2.2 | 1.1 | 0.3 | 0.3 | 0.0 | 0.1 | 0.6 | 0.2 | 0.1 | 1.0 | 6.0 |
| Elbasan AL | 0.9 | 4.0 | 1.3 | 3.2 | 2.3 | 1.1 | 0.4 | 0.4 | 0.0 | 0.1 | 0.4 | 0.2 | 0.1 | 1.1 | 6.1 |
| Tirana AL | 2.3 | 4.4 | 1.4 | 3.5 | 2.7 | 2.0 | 0.4 | 0.5 | 0.0 | 0.1 | 0.8 | 0.2 | 0.2 | 1.1 | 8.1 |
| Banja-Luka BA | 1.6 | 3.8 | 1.0 | 1.8 | 2.2 | 0.9 | 0.4 | 0.3 | 0.0 | 0.1 | 0.5 | 0.1 | 0.1 | 1.8 | 6.4 |
| Sarajevo BA | 0.5 | 2.7 | 1.8 | 2.3 | 2.2 | 0.8 | 0.2 | 0.3 | 0.0 | 0.1 | 0.3 | 0.1 | 0.1 | 1.0 | 5.1 |
| Prishtine-Pristina KS | 8.2 | 3.3 | 0.8 | 2.8 | 7.2 | 2.1 | 0.2 | 0.4 | 0.1 | 0.1 | 0.3 | 0.0 | 0.2 | 1.6 | 12.3 |
| Podgorica ME | 1.0 | 4.1 | 0.5 | 2.4 | 1.9 | 1.8 | 0.2 | 0.3 | 0.1 | 0.1 | 0.4 | 0.1 | 0.1 | 0.7 | 5.6 |
| Skopje MK | 2.3 | 7.1 | 1.9 | 2.7 | 4.0 | 2.1 | 0.6 | 1.0 | 0.1 | 0.2 | 0.7 | 0.2 | 0.2 | 2.1 | 11.3 |
| Belgrade RS | 6.5 | 3.2 | 2.1 | 2.3 | 5.3 | 2.6 | 0.3 | 0.6 | 0.1 | 0.1 | 0.8 | 0.1 | 0.1 | 1.9 | 11.9 |
| Kragujevac RS | 1.6 | 4.4 | 4.2 | 2.3 | 4.0 | 2.3 | 0.3 | 0.4 | 0.0 | 0.1 | 0.6 | 0.1 | 0.2 | 2.2 | 10.3 |
| Nis RS | 2.2 | 5.8 | 2.5 | 2.6 | 3.4 | 2.8 | 0.4 | 0.5 | 0.0 | 0.1 | 0.9 | 0.1 | 0.2 | 2.1 | 10.5 |
| Novi-Sad RS | 2.8 | 5.0 | 4.1 | 2.1 | 4.7 | 2.6 | 0.4 | 0.5 | 0.0 | 0.1 | 0.8 | 0.2 | 0.2 | 2.5 | 11.9 |
| Subotica RS | 1.0 | 7.3 | 1.9 | 2.1 | 3.3 | 1.8 | 0.5 | 0.9 | 0.0 | 0.0 | 1.2 | 0.4 | 0.1 | 2.1 | 10.3 |
| Graz AT | 2.5 | 5.1 | 1.3 | 1.2 | 1.4 | 1.5 | 0.8 | 0.5 | 0.0 | 0.2 | 1.3 | 0.4 | 0.4 | 2.5 | 8.9 |
| Linz AT | 4.4 | 5.6 | 2.1 | 2.1 | 1.4 | 1.7 | 0.9 | 1.9 | 0.0 | 0.2 | 1.6 | 0.8 | 0.4 | 3.2 | 12.0 |
| Wien AT | 4.3 | 6.3 | 0.6 | 0.8 | 2.1 | 1.7 | 0.8 | 0.5 | 0.0 | 0.2 | 1.9 | 0.6 | 0.6 | 2.9 | 11.2 |
| Burgas BG | 0.8 | 4.6 | 1.5 | 5.1 | 2.0 | 0.9 | 0.5 | 1.0 | 0.0 | 0.1 | 0.3 | 0.2 | 0.2 | 1.7 | 6.9 |
| Plovdiv BG | 3.0 | 5.5 | 2.5 | 3.7 | 2.6 | 2.4 | 0.8 | 1.3 | 0.1 | 0.1 | 0.6 | 0.1 | 0.5 | 2.4 | 10.9 |
| Sofia BG | 5.0 | 5.5 | 1.4 | 3.3 | 2.8 | 2.9 | 1.0 | 1.5 | 0.0 | 0.1 | 0.7 | 0.1 | 0.6 | 2.0 | 11.8 |
| Varna BG | 1.8 | 4.0 | 1.0 | 5.2 | 2.6 | 0.8 | 0.4 | 0.7 | 0.0 | 0.1 | 0.3 | 0.1 | 0.3 | 1.4 | 6.7 |
| Brno CZ | 1.6 | 7.1 | 2.2 | 1.0 | 1.9 | 2.0 | 0.6 | 0.4 | 0.0 | 0.1 | 1.2 | 0.4 | 0.3 | 3.9 | 10.8 |
| Liberec CZ | 0.8 | 7.3 | 2.0 | 0.9 | 2.3 | 1.4 | 0.4 | 0.3 | 0.0 | 0.1 | 0.9 | 0.3 | 0.3 | 4.2 | 10.2 |
| Ostrava CZ | 2.5 | 9.2 | 0.7 | 1.7 | 2.8 | 2.9 | 0.5 | 0.7 | 0.0 | 0.1 | 1.0 | 0.4 | 0.4 | 3.7 | 12.4 |
| Plzen CZ | 2.1 | 6.2 | 1.7 | 0.8 | 2.3 | 1.3 | 0.5 | 0.4 | 0.0 | 0.1 | 1.0 | 0.4 | 0.3 | 3.8 | 10.1 |
| Praha CZ | 4.2 | 6.6 | 1.9 | 0.6 | 2.4 | 2.5 | 0.7 | 0.5 | 0.0 | 0.1 | 1.5 | 0.6 | 0.3 | 4.2 | 12.7 |
| Usti nad Labem CZ | 1.2 | 8.1 | 3.3 | 0.8 | 3.0 | 1.6 | 0.6 | 0.7 | 0.0 | 0.2 | 1.5 | 0.6 | 0.3 | 4.0 | 12.5 |
| Zagreb HR | 3.8 | 5.5 | 1.0 | 1.2 | 1.7 | 1.7 | 0.8 | 1.2 | 0.0 | 0.1 | 1.1 | 0.4 | 0.5 | 2.6 | 10.2 |
| Budapest HU | 5.0 | 6.9 | 1.6 | 1.7 | 2.8 | 3.3 | 0.8 | 1.2 | 0.0 | 0.1 | 1.5 | 0.3 | 0.9 | 2.6 | 13.5 |
| Bucuresti RO | 7.5 | 4.3 | 4.9 | 3.8 | 3.0 | 4.1 | 1.7 | 1.7 | 0.0 | 0.1 | 1.1 | 0.1 | 0.9 | 4.0 | 16.7 |
| Iasi RO | 2.2 | 2.4 | 2.9 | 5.7 | 1.7 | 2.0 | 0.5 | 0.5 | 0.0 | 0.0 | 0.5 | 0.1 | 0.3 | 1.9 | 7.5 |
| Ljubljana SI | 6.1 | 5.3 | 1.8 | 0.9 | 2.8 | 3.1 | 0.6 | 0.5 | 0.0 | 0.2 | 1.9 | 0.4 | 0.5 | 3.0 | 13.1 |
| Bratislava SK | 5.6 | 8.5 | 1.4 | 1.1 | 2.6 | 3.9 | 1.0 | 1.3 | 0.0 | 0.1 | 1.5 | 0.4 | 1.4 | 3.4 | 15.6 |
| Kosice SK | 2.6 | 7.4 | 1.5 | 2.9 | 2.9 | 3.0 | 0.7 | 0.7 | 0.0 | 0.0 | 0.9 | 0.3 | 0.3 | 2.9 | 11.6 |

***Table S4. Combined impacts (%) of the areas and SNAP macrosectors on the $PM_{2.5}$ concentrations of the 33 studied cities.***

| GEOGRAPHIC SOURCE | SNAP MACROSECTOR | Durres AL | Elbasan AL | Tirana AL | Banja-Luka BA | Sarajevo BA | Prishtine-Pristina KS | Podgorica ME | Skopje MK | Belgrade RS | Kragujevac RS | Nis RS | Novi-Sad RS | Subotica RS | Graz AT | Linz AT | Wien AT | Burgas BG | Plovdiv BG | Sofia BG | Varna BG | Brno CZ | Liberec CZ | Ostrava CZ | Plzen CZ | Praha CZ | Usti nad Labem CZ | Zagreb HR | Budapest HU | Bucuresti RO | Iasi RO | Ljubljana SI | Bratislava SK | Kosice SK |
|---|---|---|---|---|---|---|---|---|---|---|---|---|---|---|---|---|---|---|---|---|---|---|---|---|---|---|---|---|---|---|---|---|---|---|
| FUA | 1 | 0.0 | 0.0 | 3.0 | 6.1 | 0.0 | 42.6 | 0.0 | 0.3 | 25.6 | 1.7 | 0.0 | 5.8 | 0.0 | 2.2 | 1.6 | 4.6 | 0.2 | 0.7 | 1.5 | 10.0 | 0.3 | 0.8 | 2.4 | 8.6 | 5.3 | 2.0 | 1.7 | 1.8 | 1.3 | 2.9 | 12.8 | 1.7 | 4.2 |
| FUA | 2 | 1.3 | 2.6 | 6.9 | 2.9 | 2.7 | 6.6 | 8.6 | 4.1 | 10.0 | 6.0 | 8.6 | 7.0 | 2.0 | 4.3 | 3.2 | 4.6 | 2.0 | 7.3 | 11.4 | 1.2 | 4.8 | 2.4 | 3.7 | 4.7 | 10.7 | 2.1 | 6.3 | 10.8 | 11.0 | 6.3 | 12.3 | 12.2 | 6.5 |
| FUA | 3 | 0.1 | 0.4 | 0.4 | 1.5 | 0.1 | 0.2 | 0.1 | 1.0 | 0.3 | 0.1 | 0.1 | 0.1 | 0.0 | 4.2 | 3.2 | 2.8 | 0.5 | 2.2 | 4.2 | 0.2 | 0.9 | 0.7 | 0.6 | 1.6 | 2.2 | 0.6 | 3.8 | 2.1 | 5.3 | 1.3 | 2.0 | 2.3 | 1.2 |
| FUA | 4 | 0.2 | 1.4 | 2.2 | 0.7 | 1.0 | 1.6 | 0.5 | 3.9 | 2.6 | 0.2 | 0.4 | 0.8 | 3.2 | 1.7 | 10.4 | 1.4 | 1.6 | 3.5 | 6.9 | 0.4 | 0.4 | 0.4 | 1.6 | 0.6 | 1.3 | 0.6 | 7.7 | 5.1 | 5.0 | 1.1 | 1.3 | 4.8 | 1.5 |
| FUA | 5 | 0.1 | 0.1 | 0.2 | 0.2 | 0.1 | 0.4 | 0.2 | 0.4 | 0.3 | 0.1 | 0.1 | 0.1 | 0.0 | 0.0 | 0.0 | 0.0 | 0.0 | 0.4 | 0.1 | 0.0 | 0.0 | 0.0 | 0.1 | 0.0 | 0.0 | 0.0 | 0.2 | 0.0 | 0.0 | 0.0 | 0.0 | 0.1 | 0.0 |
| FUA | 6 | 0.2 | 0.4 | 0.7 | 0.4 | 0.4 | 0.3 | 0.2 | 0.7 | 0.6 | 0.2 | 0.2 | 0.2 | 0.1 | 1.3 | 0.9 | 1.1 | 0.1 | 0.4 | 0.5 | 0.1 | 0.2 | 0.3 | 0.5 | 0.3 | 0.4 | 0.3 | 0.8 | 0.4 | 0.6 | 0.1 | 0.6 | 0.2 | 0.1 |
| FUA | 7 | 1.3 | 1.0 | 3.9 | 1.5 | 1.5 | 0.8 | 1.4 | 2.7 | 3.8 | 1.5 | 2.9 | 1.7 | 0.7 | 4.4 | 3.6 | 8.3 | 0.5 | 1.4 | 3.3 | 0.4 | 1.4 | 0.9 | 1.5 | 1.6 | 4.8 | 1.3 | 3.6 | 4.5 | 2.5 | 1.2 | 7.0 | 2.3 | 0.6 |
| FUA | 8 | 0.4 | 1.0 | 0.2 | 0.1 | 0.0 | 0.0 | 0.1 | 0.6 | 0.3 | 0.1 | 0.2 | 0.2 | 0.1 | 1.7 | 3.5 | 2.9 | 0.9 | 0.1 | 0.2 | 0.4 | 0.7 | 0.3 | 0.8 | 1.0 | 2.0 | 0.9 | 1.9 | 0.7 | 0.2 | 0.2 | 1.0 | 0.5 | 0.1 |
| FUA | 9 | 0.2 | 0.2 | 1.0 | 0.3 | 0.4 | 0.3 | 0.0 | 0.4 | 0.1 | 0.2 | 0.2 | 0.2 | 0.1 | 2.3 | 1.1 | 3.7 | 0.3 | 1.1 | 2.9 | 1.6 | 1.1 | 0.4 | 1.1 | 0.4 | 1.0 | 0.2 | 2.9 | 4.6 | 3.1 | 1.4 | 2.0 | 7.1 | 0.6 |
| FUA | 10 | 1.4 | 2.1 | 2.0 | 5.5 | 1.1 | 1.6 | 1.0 | 2.1 | 2.9 | 3.1 | 3.8 | 3.8 | 2.0 | 2.9 | 3.3 | 6.3 | 0.7 | 3.2 | 1.8 | 0.5 | 3.9 | 1.4 | 5.4 | 0.9 | 3.7 | 0.7 | 4.1 | 2.7 | 7.4 | 2.0 | 4.4 | 2.8 | 3.1 |
| NATIONAL | 1 | 0.5 | 0.6 | 0.1 | 7.7 | 16.2 | 0.0 | 1.3 | 7.3 | 4.1 | 15.8 | 6.2 | 14.8 | 8.0 | 0.9 | 1.3 | 0.3 | 6.4 | 4.6 | 3.3 | 2.5 | 2.1 | 3.7 | 0.6 | 5.7 | 4.2 | 9.1 | 1.3 | 0.9 | 2.8 | 1.5 | 2.2 | 0.5 | 0.6 |
| NATIONAL | 2 | 3.4 | 3.9 | 4.3 | 0.9 | 1.7 | 3.1 | 2.5 | 1.3 | 4.2 | 6.7 | 5.0 | 5.3 | 2.7 | 1.6 | 1.7 | 0.5 | 1.4 | 3.5 | 1.9 | 1.1 | 2.4 | 2.3 | 0.7 | 2.0 | 1.9 | 3.2 | 1.1 | 1.9 | 6.2 | 6.3 | 3.7 | 2.6 | 4.0 |
| NATIONAL | 3 | 0.6 | 0.9 | 0.7 | 0.5 | 0.8 | 0.3 | 0.1 | 0.8 | 0.3 | 0.4 | 0.3 | 0.7 | 0.3 | 0.8 | 1.1 | 0.3 | 0.3 | 0.9 | 0.4 | 0.4 | 0.8 | 0.7 | 0.2 | 0.7 | 0.7 | 1.1 | 0.5 | 0.3 | 1.2 | 1.0 | 0.5 | 0.6 | 0.6 |
| NATIONAL | 4 | 0.5 | 0.4 | 0.4 | 0.4 | 0.8 | 0.1 | 0.3 | 0.6 | 0.4 | 0.9 | 0.5 | 0.8 | 0.4 | 0.4 | 0.8 | 0.1 | 0.6 | 1.6 | 0.7 | 0.6 | 0.4 | 0.5 | 0.2 | 0.6 | 0.7 | 2.2 | 1.1 | 0.7 | 1.2 | 1.0 | 0.3 | 0.7 | 0.6 |
| NATIONAL | 5 | 0.2 | 0.2 | 0.1 | 0.1 | 0.2 | 0.1 | 0.3 | 0.2 | 0.1 | 0.2 | 0.1 | 0.1 | 0.0 | 0.0 | 0.0 | 0.0 | 0.0 | 0.0 | 0.0 | 0.0 | 0.0 | 0.0 | 0.0 | 0.0 | 0.0 | 0.0 | 0.0 | 0.0 | 0.0 | 0.0 | 0.0 | 0.0 | 0.0 |
| NATIONAL | 6 | 0.5 | 0.4 | 0.4 | 0.1 | 0.3 | 0.1 | 0.1 | 0.2 | 0.2 | 0.2 | 0.2 | 0.2 | 0.1 | 0.2 | 0.3 | 0.0 | 0.1 | 0.1 | 0.0 | 0.1 | 0.1 | 0.1 | 0.1 | 0.1 | 0.1 | 0.3 | 0.1 | 0.0 | 0.1 | 0.1 | 0.1 | 0.0 | 0.0 |
| NATIONAL | 7 | 1.5 | 1.4 | 1.0 | 0.4 | 0.6 | 0.2 | 0.4 | 0.4 | 0.6 | 1.4 | 1.1 | 1.2 | 0.8 | 2.8 | 3.0 | 1.3 | 0.5 | 1.1 | 0.5 | 0.5 | 1.6 | 1.8 | 0.4 | 1.7 | 1.5 | 2.9 | 0.9 | 1.2 | 2.1 | 1.6 | 1.8 | 0.8 | 0.9 |
| NATIONAL | 8 | 0.6 | 0.7 | 0.6 | 0.0 | 0.0 | 0.0 | 0.0 | 0.5 | 0.1 | 0.1 | 0.1 | 0.1 | 0.1 | 0.6 | 0.8 | 0.3 | 0.3 | 0.1 | 0.1 | 0.2 | 0.9 | 0.8 | 0.2 | 0.9 | 0.8 | 1.4 | 0.3 | 0.3 | 0.2 | 0.2 | 0.4 | 0.1 | 0.1 |
| NATIONAL | 9 | 0.3 | 0.4 | 0.4 | 0.1 | 0.2 | 0.3 | 0.1 | 0.1 | 0.3 | 0.4 | 0.3 | 0.3 | 0.2 | 0.5 | 0.5 | 0.2 | 1.0 | 1.8 | 0.5 | 1.0 | 0.4 | 0.4 | 0.1 | 0.2 | 0.2 | 0.3 | 0.3 | 0.4 | 0.6 | 0.6 | 0.4 | 0.2 | 0.3 |
| NATIONAL | 10 | 3.7 | 4.6 | 3.9 | 1.9 | 3.7 | 1.4 | 1.2 | 2.4 | 4.7 | 7.5 | 5.7 | 5.9 | 3.1 | 5.3 | 5.1 | 2.4 | 1.9 | 3.0 | 1.9 | 1.8 | 9.5 | 8.0 | 2.7 | 4.2 | 4.4 | 4.3 | 3.0 | 4.9 | 9.6 | 9.8 | 3.1 | 2.8 | 3.4 |
| INTERNATIONAL | 1 | 21.6 | 23.5 | 20.0 | 13.2 | 13.5 | 5.4 | 21.9 | 20.9 | 7.6 | 14.2 | 20.1 | 13.0 | 18.4 | 10.4 | 7.2 | 12.3 | 10.5 | 12.8 | 13.8 | 9.2 | 13.7 | 16.2 | 16.7 | 6.9 | 8.4 | 11.8 | 11.5 | 15.7 | 10.3 | 8.1 | 5.4 | 13.3 | 15.1 |
| INTERNATIONAL | 2 | 6.8 | 5.8 | 6.4 | 7.3 | 6.3 | 4.3 | 11.3 | 9.6 | 4.4 | 5.9 | 7.5 | 6.1 | 10.1 | 9.3 | 6.8 | 8.7 | 4.6 | 5.4 | 5.9 | 4.2 | 9.7 | 7.8 | 15.9 | 5.7 | 5.9 | 7.0 | 8.0 | 8.9 | 2.8 | 2.7 | 6.2 | 8.7 | 10.4 |
| INTERNATIONAL | 3 | 2.7 | 2.6 | 2.3 | 2.5 | 1.9 | 0.8 | 2.8 | 2.4 | 1.2 | 2.1 | 2.8 | 2.1 | 4.0 | 2.5 | 2.0 | 3.2 | 3.7 | 2.7 | 2.0 | 3.1 | 3.3 | 2.2 | 2.7 | 2.3 | 2.0 | 2.7 | 2.5 | 3.0 | 1.8 | 1.7 | 1.9 | 2.8 | 2.9 |
| INTERNATIONAL | 4 | 2.3 | 2.0 | 2.0 | 2.6 | 1.8 | 1.3 | 2.7 | 2.4 | 1.3 | 1.9 | 2.6 | 1.8 | 3.3 | 2.6 | 2.0 | 2.5 | 5.9 | 3.7 | 2.5 | 4.4 | 2.6 | 2.1 | 2.9 | 2.4 | 2.0 | 2.6 | 2.0 | 2.4 | 1.9 | 1.5 | 2.0 | 2.3 | 2.4 |
| INTERNATIONAL | 5 | 0.2 | 0.2 | 0.1 | 0.1 | 0.1 | 0.1 | 0.2 | 0.2 | 0.1 | 0.1 | 0.1 | 0.1 | 0.1 | 0.1 | 0.1 | 0.1 | 0.0 | 0.1 | 0.1 | 0.0 | 0.1 | 0.1 | 0.2 | 0.1 | 0.1 | 0.1 | 0.1 | 0.1 | 0.0 | 0.0 | 0.1 | 0.0 | 0.1 |
| INTERNATIONAL | 6 | 0.4 | 0.2 | 0.2 | 0.4 | 0.2 | 0.3 | 0.6 | 0.5 | 0.2 | 0.2 | 0.2 | 0.1 | 0.2 | 0.4 | 0.5 | 0.3 | 0.3 | 0.3 | 0.2 | 0.3 | 0.2 | 0.3 | 0.5 | 0.4 | 0.2 | 0.7 | 0.3 | 0.1 | 0.0 | 0.0 | 0.7 | 0.1 | 0.1 |
| INTERNATIONAL | 7 | 2.9 | 2.3 | 1.7 | 4.2 | 2.3 | 0.8 | 2.9 | 2.1 | 1.4 | 2.2 | 2.8 | 3.0 | 8.0 | 5.2 | 4.4 | 6.1 | 1.4 | 1.2 | 0.9 | 1.5 | 7.1 | 5.2 | 4.9 | 6.2 | 4.7 | 7.2 | 5.4 | 4.0 | 0.9 | 0.9 | 5.0 | 6.1 | 4.6 |
| INTERNATIONAL | 8 | 1.0 | 0.9 | 0.7 | 1.4 | 0.8 | 0.3 | 1.0 | 0.6 | 0.5 | 0.7 | 0.9 | 1.0 | 2.6 | 1.6 | 1.6 | 2.1 | 0.4 | 0.5 | 0.4 | 0.4 | 2.1 | 1.7 | 1.5 | 1.7 | 1.4 | 2.1 | 1.5 | 1.3 | 0.3 | 0.3 | 1.6 | 2.1 | 1.7 |
| INTERNATIONAL | 9 | 0.5 | 0.5 | 0.5 | 1.0 | 0.7 | 0.5 | 0.9 | 1.0 | 0.5 | 0.6 | 0.7 | 0.7 | 0.9 | 1.3 | 1.0 | 1.1 | 0.3 | 0.5 | 0.6 | 0.3 | 1.3 | 1.5 | 1.7 | 1.6 | 1.3 | 1.4 | 1.1 | 0.7 | 0.4 | 0.2 | 1.2 | 1.2 | 1.0 |
| INTERNATIONAL | 10 | 5.0 | 4.6 | 3.9 | 13.8 | 9.1 | 7.8 | 6.9 | 10.9 | 5.6 | 7.0 | 6.7 | 7.9 | 11.5 | 16.6 | 14.1 | 15.6 | 11.2 | 10.1 | 9.7 | 9.6 | 19.5 | 28.8 | 18.4 | 30.0 | 23.4 | 25.0 | 16.0 | 9.1 | 2.5 | 2.9 | 13.9 | 14.5 | 13.3 |

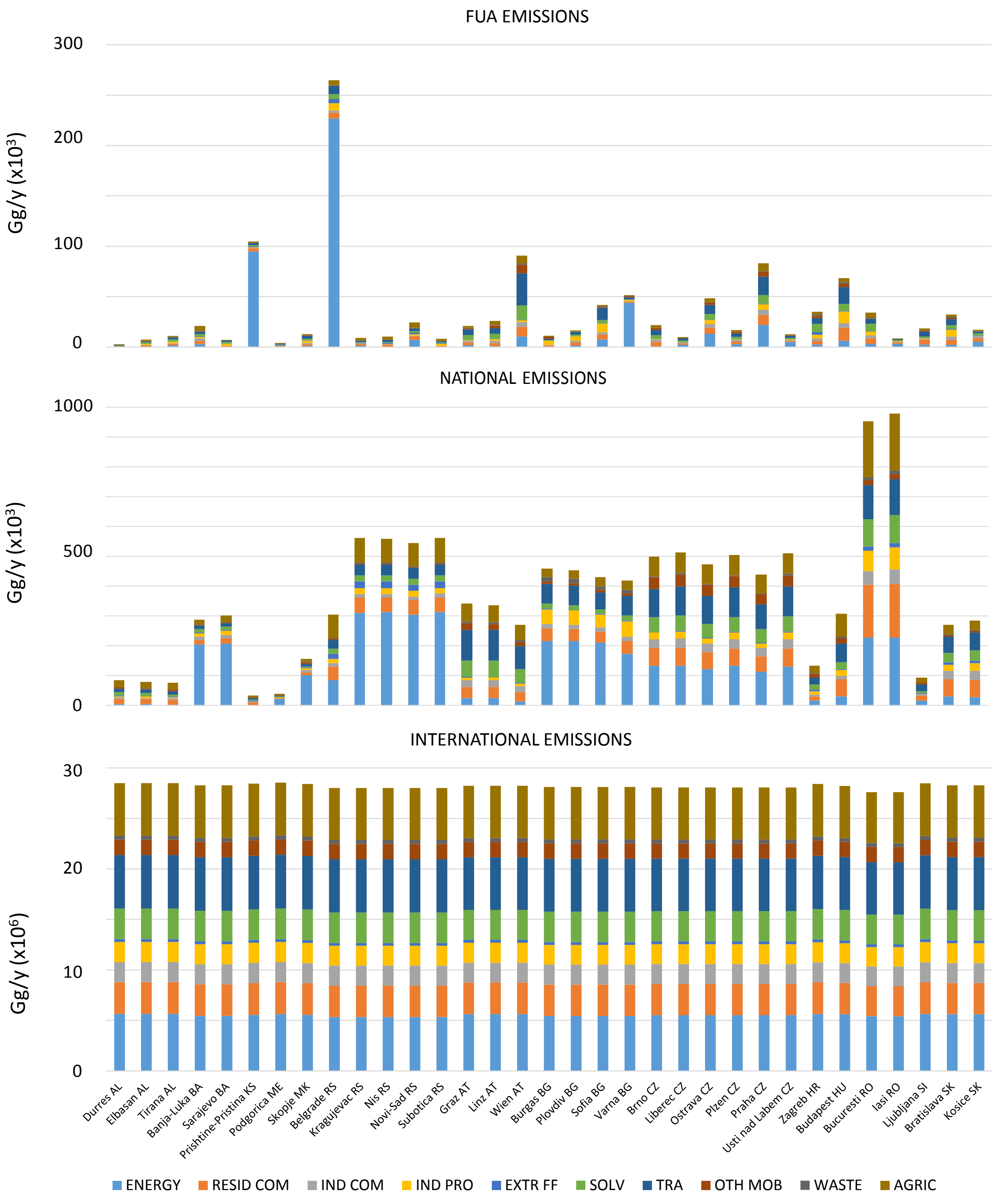


***Figure S5. Model input emissions split by geographical level and activity sectors to estimate the impact of $PM_{2.5}$ sources in every city of this study (international, representing the emission from the entire model domain, with the exception of the country itself).***

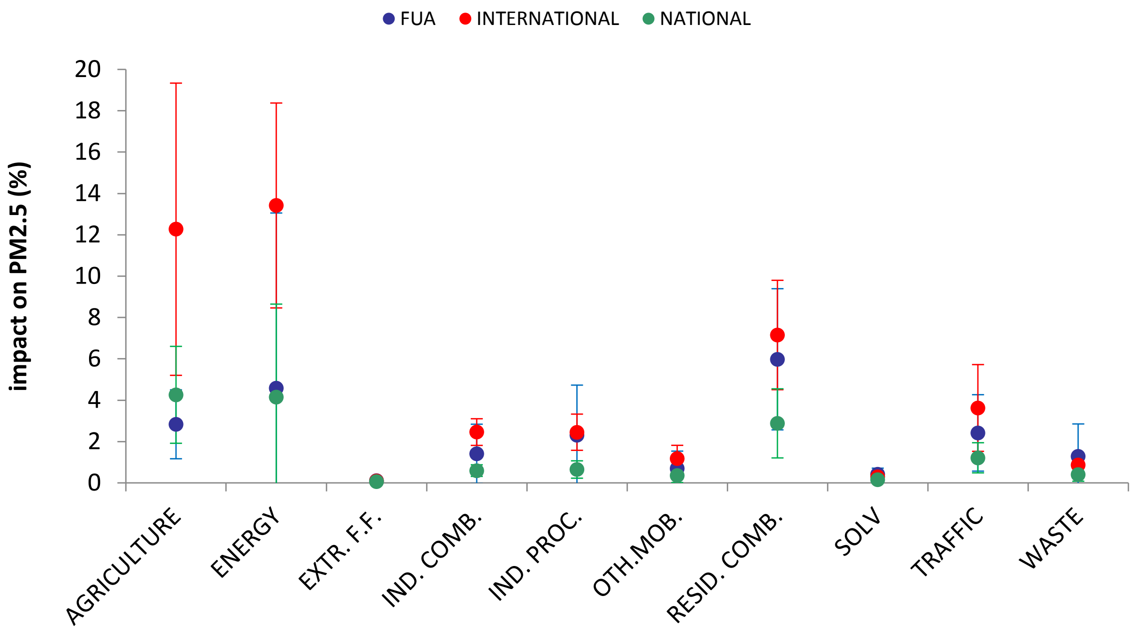


***Figure S6. Summary of the impact of the activity sources (mean and standard deviation) split by geographical origin. AGRICULTURE: agriculture, ENERGY: energy combustion, EXTR. F.F.: extraction and distribution of fossil fuels, IND COMB.: combustion in industry, IND. PRO.: industrial processes, OTH. MOB.: other mobile sources, RESID. COMB.: residential combustion, SOLV: use of solvents, TRAFFIC: road transport, WASTE: waste management.***
